# AEOL-induced NRF2 activation and DWORF overexpression mitigate myocardial I/R injury

**DOI:** 10.1186/s10020-025-01242-1

**Published:** 2025-05-15

**Authors:** Maria del Carmen Asensio-Lopez, Miriam Ruiz-Ballester, Silvia Pascual-Oliver, Francisco Jose Bastida-Nicolas, Yassine Sassi, Jose Javier Fuster, Domingo Pascual-Figal, Maria Josefa Fernandez del Palacio, Fernando Soler, Antonio Lax

**Affiliations:** 1 Centro Nacional de Investigaciones Cardiovasculares (CNIC), Madrid, Spain; 2 R&D Department, Biocardio S.L, El Palmar, Murcia, Spain; 3 Present Address: Instituto Murciano de Investigación Biosanitaria (IMIB) Pascual Parrilla and University of Murcia, Ctra. Madrid-Cartagena S/N, Murcia, Spain; 4 Fralin Biomedical Research Institute at Virginia Tech Carilion, Roanoke, Virginia USA; 5 CIBER en Enfermedades Cardiovasculares (CIBER-CV), Madrid, Spain; 6 Cardiology Department, Hospital Virgen de La Arrixaca, IMIB-Pascual Parrilla, University of Murcia, El Palmar, Murcia, Spain; 7 Veterinary Teaching Hospital, Veterinary Medicine and Surgery Department, University of Murcia, El Palmar, Murcia, Spain

**Keywords:** Acute myocardial infarction, Reperfusion injury, NRF2, DWORF, AEOL-10150, Cardiac protection

## Abstract

**Background:**

The causal relationship between the activation of nuclear factor erythroid 2-related factor 2 (NRF2) and the preservation of SERCA2a function in mitigating myocardial ischemia–reperfusion (mI/R) injury, along with the associated regulatory mechanisms, remains incompletely understood. This study aims to unravel how NRF2 directly or indirectly influences SERCA2a function and its regulators, phospholamban (PLN) and Dwarf Open Reading Frame (DWORF), by testing the pharmacological repositioning of AEOL-10150 (AEOL) in the context of mI/R injury.

**Methods:**

C57BL6/J, *Nrf2* knockout (*Nrf2*^*−/−*^), and wild-type (*Nrf2*^+*/*+^) mice, as well as human induced pluripotent stem cell-derived cardiomyocytes (hiPSCMs) were subjected to I/R injury. Gain/loss of function techniques, RT-qPCR, western blotting, LC/MS/MS, and fluorescence spectroscopy were utilized. Cardiac dimensions and function were assessed by echocardiography.

**Results:**

In the early stages of mI/R injury, AEOL administration reduced mitochondrial ROS production, decreased myocardial infarct size, and improved cardiac function. These effects were due to NRF2 activation, leading to the overexpression of the micro-peptide DWORF, consequently enhancing SERCA2a activity. The cardioprotective effect induced by AEOL was diminished in *Nrf2*^*−/−*^ mice and in *Nrf2*/*Dworf* knockdown models in hiPSCMs subjected to simulated I/R injury. Our data show that AEOL-induced NRF2-mediated upregulation of DWORF disrupts the phospholamban-SERCA2a interaction, leading to enhanced SERCA2a activation and improved cardiac function.

**Conclusions:**

Taken together, our study reveals that AEOL-induced NRF2-mediated overexpression of DWORF enhances myocardial function through the activation of the SERCA2a offering promising therapeutic avenues for mI/R injury.

**Supplementary Information:**

The online version contains supplementary material available at 10.1186/s10020-025-01242-1.

## Highlights

• *Novel AEOL-10150 therapeutic potential.* AEOL-10150 demonstrates promise in activating NRF2 and mitigating myocardial ischemia-reperfusion injury.

• *DWORF overexpression breakthrough.* Overexpression of DWORF significantly contributes to preserving cardiac function and reducing myocardial injury through the NRF2-DWORF pathway.

• *Enhanced cardiac protection mechanisms.* The study highlights the dual role of AEOL-10150 and DWORF in enhancing cardiac protection and preventing heart failure.

• *Future research directions.* Additional studies are required to validate the long-term efficacy of AEOL-10150 and the regulatory effects of NRF2-DWORF axis in clinical applications.

**Supplementary Information:**

The online version contains supplementary material available at 10.1186/s10020-025-01242-1.

## Introduction

Acute myocardial infarction (AMI), triggered by thrombotic occlusion of a coronary artery, is a leading cause of morbidity and disability worldwide (Tsao et al. 2022). Current treatment strategies for AMI rely on percutaneous coronary intervention (PCI) to rapidly restore coronary artery blood flow. Unfortunately, this reperfusion procedure can itself trigger profound myocardial injury, additional to that caused by the preceding ischemia, and the compound effects of this myocardial ischemia–reperfusion (mI/R) injury include cardiomyocyte death, cardiac dysfunction, and ventricular remodeling (Miura and Miki 2008; Eltzschig et al. 2011; Liang et al. 2017; Aldakkak et al. 2011). An analysis of AMA data from 2008 showed that, despite timely PCI, approximately 10% of AMI patients died during their index hospitalization and 25% of survivors developed chronic heart failure (CHF) (Roger et al. 2012). Current treatments for CHF, including vasodilators, loop diuretics, and inotropic agents, help to alleviate congestion and improve hemodynamics in the short-term. However, there is a lack of drugs able to prevent end-organ damage or improve long-term outcomes in AMI patients treated by PCI (Krum and Teerlink 2011). It is therefore vital to explore novel therapeutic options to alleviate ventricular remodeling and prevent progression to CHF.

Extensive research over four decades has shown that mI/R-injury is mediated by two major mechanisms, oxidative damage and cytosolic Ca^2+^overload (Gutierrez et al. 2006; Takimoto et al. 2007; Santos et al. 2011). A crucial role in these processes is played by nuclear factor erythroid 2-related factor 2 (NRF2), a key transcription factor in the cellular antioxidant response (Shen et al. 2019). NRF2 regulates the expression of a series of genes involved in defense against oxidative damage, including antioxidant enzymes and detoxifying proteins (Chen and Maltagliati 2018). NRF2 activation can counteract mitochondrial oxidative damage (Chen 2022) and preserve the function of essential proteins such as sarcoplasmic reticulum (SR) Ca^2+^-ATPase (SERCA2a) (Wang 2022). SERCA2a, whose activity is regulated by the accessory protein phospholamban (PLN) and by a recently discovered SR localized micropeptide termed Dwarf Open Reading Frame (DWORF) (Weber et al. 2021; Fisher et al. 2021), plays a key role in cardiac muscle by pumping Ca^2+^from the cytosol to the SR, directly influencing heart contraction and relaxation (Sitsel et al. 2019). PLN acts as a natural SERCA2a inhibitor, whereas DWORF activates SERCA2a by binding to it and displacing PLN (Fisher et al. 2021). Disturbance of SERCA2a function by PLN, particularly under conditions of oxidative stress (D’Oria 2020), resulting in contractile dysfunction and intracellular Ca^2+^dysregulation (Brown 2023)which are initial events in HF (Kranias and Hajjar 2012). Targeting the PLN/SERCA2a interaction with conventional small-molecule drugs has proven challenging, thus leaving the PLN/SERCA2a axis as an unmet therapeutic promise (Fry 2015). Conversely, DWORF is significantly downregulated in humans and mice with CHF (Li et al. 2021; Fisher et al. 2021;, Nelson et al. 2016). Its overexpression has been linked to cardioprotection in both genetically induced and common forms of CHF in animal models (Makarewich 2018; Makarewich et al. 2020), likely through enhanced Ca^2+^reuptake. Furthermore, although there is substantial evidence for a functional connection between SERCA2a and NRF2, the mechanism of this relationship remains incompletely understood. Therefore, further characterization is needed to elucidate how NRF2 directly or indirectly influences SERCA2a function, as well as the roles played by PLN and DWORF in this setting. Note that, NRF2 activation holds promise as an innovative approach to cardiac protection (Chen and Maltagliati 2018).

The metalloporphyrin AEOL-10150 (AEOL), a mimic of the catalytic site of superoxide dismutase, provides protection against radiation-induced lung injury (Cui et al. 2021)and enhances myocardial function in mice undergoing anthracycline-based chemotherapy (Kliment et al. 2009). The protective role of AEOL appears to involve multiple mechanisms of action, including antioxidant (Zhang 2018), anti-inflammatory (Garofalo et al. 2014), and anti-fibrotic activities, indicating a potential therapeutic benefit in mI/R injury. Here, we assessed the impact of AEOL on NRF2 and its role as a modulator of SERCA2a activity via PLN and DWORF during mI/R injury.

## Methods

### Preparation of human iPS-derived cardiomyocytes (hiPSCMs) and simulated I/R model

Human induced pluripotent stem cells (hiPSCs) were purchased from Phenocell (PCi-CAU); ~ 0.5 × 10^5^ viable cells were provided in cryovials. hiPSCs were initially grown to 80% confluence in mTeSR™ plus medium on Matrigel® matrix to allow attachment of cell aggregates and maintained in a humidified 95% air, 5% CO_2_ atmosphere at 37 °C. When the cells reached passage 20, the reprogramming procedure was started using our standardized protocol. Briefly, the culture medium was removed and replaced with RPMI-1640 containing B27 supplement minus insulin (basal differentiation supplement) and 4 μM CHIR99021 (day 0). On day 3, the medium was changed to basal differentiation supplement containing 3 μM of IWR-1. On day 5, the medium was refreshed with basal differentiation supplement. The medium was replaced on day 8 with fresh RPMI 1640 containing B27 supplement minus insulin. On day 11, the medium was changed to glucose-free RPMI 1640 containing B27 supplement minus insulin. On day 14, the medium was reverted to RPMI 1640 containing B27 supplement minus insulin. Differentiation cultures were maintained in a 95% air, 5% CO_2_atmosphere at 37ºC. During differentiation, the medium was replaced every 3 days. Beating clusters were observed after 20 days. hiPSCMs were allowed to recover for 12 days in iCell Maintenance Medium (Cellular Dynamics International) before experiments. Before assays, standardized RT-qPCR gene expression profiles were determined for hiPSCs and derived hiPSCMs (Figure S1). OCT4 and NANOG were used as hiPSC-specific markers and cTnT and NKX 2–5 as hiPSCM-specific markers. Multiple rounds of differentiation and the procurement of additional control hiPSC-CM lines were conducted throughout the study to serve as biological replicates, ensuring the control of the cellular phenotype and that the assay was not performed with a single differentiated line. The I/R model was adapted with modifications from the experimental design of Sebastião et al. (Sebastião 2019). In brief, ischemia was mimicked by replacing iCell medium with ischemia-mimetic solution (140 mM NaCl,12 mM KCl, 1.2 mM MgCl_2_, 20 mM HEPES,1.8 mM CaCl_2_, 20 mM sodium lactate, pH 6.22) and placing the hiPSCMs cultures in a hypoxia chamber (STEMCELL Technologies; 27,310) at 37 °C in a nitrogen-enriched atmosphere to achieve a 1% O_2_ concentration. After 45 min of simulated ischemia, reperfusion was mimicked by reoxygenation in control culture conditions (iCell medium at 21% O_2_), which were maintained for a recovery period of 24 h. Control cultures were maintained throughout experiments in iCell medium at 21% O_2_. When indicated, 50 µM AEOL or 30 nM DWORF were added at the onset of reoxygenation. At the end of the 24 h reoxygenation–recovery period, cells were processed for subsequent analysis. For succinate treatment, hiPSCMs were incubated in assay buffer (132 mN NaCl, 10 mM HEPES, 4.2 mM KCl, 1 mM MgCl_2_, 1.8 mM CaCl_2_, 2.5 µM 2-deoxyglucose, 1.14 sodium pyruvate, pH 7.4) and treated with the indicated agents in the presence or absence of 5 mM dimethyl succinate or 4 μM oligomycin for 2 h. Selected AEOL concentration (50µM) was tested in cardiomyocytes under normoxia and showed no deleterious effect on the evaluated parameters (data not shown).

### Animal care and ethics

Male C57Bl/6 J mice (25–30 g) were purchased from the ENVIGO Laboratory. Colonies of Nfe2 l2 tm1Ywk (*Nrf2*) knockout mice (*Nrf2*-KO) and *Nrf2*-WT littermates were from the Jackson Laboratory. *Nrf2* gene knockout was confirmed by genotyping with the Jackson Laboratory-recommended protocol and primers: *Nrf2* common_forward (5´ -GCCTGAGAGCTGTAGGCCC-3´), *Nrf2* WT_reverse (5´-GGAATGGAAAATAGCTCCTGCC-3´), and *Nrf2* Mut_reverse (5´-GACAGTATCGGCCTCAGGAA-3´). Animals were housed in a specific pathogen-free environment at 23 ± 2 °C and 50 ± 5% relative humidity and with a 12 h light–dark cycle. Mice had free access to food and water. All animal experiments were approved by the Ethics Review committee for animal use at the University of Murcia (approval No. A13220701). Animals were adapted to the environment for 7 days before experimentation.

### In vivomyocardial I/R model and experimental design

Mice were anesthetized and ventilated via tracheal intubation with a Harvard rodent respirator. The left anterior descending coronary artery was ligated with a 6–0 silk suture slip knot positioned approximately 2 mm below the edge of the left atrial appendage. mI/R injury was initiated by tightening the slip knot to induce ischemia, followed after 45 min by release of the knot to produce myocardial reperfusion and recovery for 30 min, 24 h, 28 days (4 weeks), or 56 days (8 weeks). The animals were randomly assigned to receive AEOL (25 mg/kg in physiological saline solution (0.9% NaCl)) or vehicle (saline solution only), administered via subcutaneous injection after reperfusion onset. The AEOL dose was based on a published analysis of in vivo efficacy in mice (Murigi et al. 2015), which was tested in control animals (without mI/R) and showed no effect on the evaluated parameters (data not shown). AEOL was selected to be administered immediately after reperfusion to target the oxidative damage that begins in the acute phase, a time point shown to be critical in other non-cardiovascular models of oxidative injury (Zhang 2018; Garofalo et al. 2014). The study comprised two distinct phases. The initial phase aimed to examine the protective effects of AEOL against acute-phase mI/R injury (Figure S2, Experiment 1). Mice subjected to mI/R received a single dose of AEOL or vehicle at 15 min after reperfusion onset and were then allowed to recover for either 30 min or 24 h (Figure S2; Experiment 1). The second part of the study explored the long-term benefits of AEOL on adverse cardiac remodeling. A separate cohort of mice underwent mI/R and received daily repeat injections of AEOL or vehicle for 5 days, starting at 5 min, 15 min, or 3 days after reperfusion onset (Figure S2; Experiment 2). These mice were allowed to recover for either 4 or 8 weeks. When indicated, mice received intraperitoneal injections of 30 nM DWORF coincident with AEOL treatment.

### Measurement of mitochondrial ROS in vitro and in vivo

To measure mitochondrial ROS production in hiPSCMs, 3 × 10^6^ cells suspended in DPBS were incubated for 20 min at 25ºC with the fluorescent probe MitoSox (Invitrogen) at 5 µM. The cells were then washed and centrifuged (480 xg, 10 min), and changes in fluorescence intensity were measured at λex = 510 nm and λem = 580 nm in a Clariostar microplate reader (BMG Labtech). Mitochondrial ROS production was plotted as the relative increase in fluorescence.

Mitochondrial ROS production in the hearts of mice subjected to mI/R injury was estimated from the conversion of the ratiometric probe MitoB to MitoP (Salin et al. 2017). Briefly, 25 nmol MitoB (Cayman Chemical; 17,116) in 100 μL DPBS was administered via tail vein injection 4 h before initiating the mI/R protocol (with or without AEOL injection 15 min after reperfusion onset). After reperfusion and recovery for 30 min or 24 h, hearts were removed. The infarct border zone was processed and flash frozen in liquid nitrogen. Heart tissue samples were homogenized, spiked with deuterated internal standards [d_15_-MitoB (Cayman Chemical; 17,470) and d_15_-MitoP (Cayman Chemical; 19,296)], and MitoB and its product MitoP were determined by liquid chromatography and tandem mass spectrometry. The MitoP/MitoB ratio was plotted as the relative increase relative to sham operated mice.

### Measurement of oxidative DNA damage

DNA samples were prepared from the infarct border zone 30 min after mI/R, incubated at 95ºC for 5 min, and rapidly chilled on ice to prevent re-annealing of single-stranded DNA. The DNA was then digested with 5 units of nuclease P1 in 20 mM sodium acetate, pH 5.2 for 2 h at 37ºC. Next, samples were resuspended in 100 mM Tris buffer, pH 7.5 and treated with 5 units of alkaline phosphatase for 1 h at 37ºC. The reaction mixtures were centrifuged for 5 min at 6,000 × g, and the supernatants were analyzed by competitive ELISA for 8-hydroxy-2’-deoxy guanosine (8-OHdG) (OxiSelect™ Oxidative DNA Damage ELISA Kit; Cell Biolabs STA-320-T). Absorbance was read at 450 nm in a Clariostar microplate reader (BMG Labtech), and the fold change in 8-OHdG was plotted relative to sham-operated mice.

### Mitochondrial function and cell viability

hiPSCMs were subjected to simulated I/R with or without AEOL treatment at the onset of reoxygenation. At the end of the 24-h reoxygenation period, the conditioned medium was collected for lactate assay (Abcam; ab653331), and the cells were harvested for the determination of ATP content with the ATP Luminometric Assay kit (Beyotime Institute of Biotechnology). Total protein was determined by the bicinchoninic acid (BCA) method (Smith et al. 1985). To measure mitochondrial membrane potential (Δψm), hiPSCMs were loaded with TMRE (500 nm) (Abcam; ab113852) for 30 min at 37 °C. Cells were then washed twice with warm DPBS containing 0.2% bovine serum albumin (BSA) (w/v), and fluorescence intensity was detected in a plate reader with excitation/emission at 549/575 nm. MPTP opening was assayed with the Mitochondrial Transition Pore Assay Kit (Life Technology). Cell viability was measured with the Cell Counting Kit-8 (Enzo; ALX-850–039). Measurements and analysis were carried out in a Clariostar microplate reader (BMG Labtech).

### TUNEL assay of mI/R-induced apoptotic cell death

In mice at 24 h post-mI/R, hearts were arrested in diastole by intravenous injection of a 0.2 mL bolus of 30% (w/v) KCl (Merck). The hearts were excised and rinsed with ice-cold DPBS before removal of the right ventricle and atria. Mid-papillary slices of the left ventricle (*n* = 7 mice per treatment group) were fixed in 4% (w/v) formaldehyde for up to 24 h before paraffin embedding. The slices were stained by terminal deoxynucleotidyl transferase dUTP nick end labeling (DeadEnd colorimetric TUNEL system, Promega Corporation). Apoptotic cells were identified by dark-brown precipitation in cardiomyocyte nuclei, visualized in high-power visual fields (400X) with an Axioscope Axio A10 brightfield microscope (Carl Zeiss) fitted with a high-resolution color digital camera (AxioCam 506 color). Representative images were obtained with Zeiss Zen, version 3.0 (Cals Zeiss).

### Assessment of myocardial infarct size

Mice at 8 weeks post-mI/R were given a KCl bolus as above to arrest the heart in diastole. After sacrifice, hearts were excised, rinsed in ice-cold DPBS, and frozen by immersion in liquid nitrogen for 10 min. Frozen mid-papillary slices (1–3 mm) of the LV of seven mice from each treatment group were immersed in 1% 2,3,5-triphenyl-tetrazolium chloride (TTC; Merck) in DPBS at 37 °C for 15 min to stain the non-infarcted tissue. The slices were photographed under standardized lighting to optimize contrast and highlight viable tissue, with consistent brightness across all images. No post-hoc alterations, such as color correction or contrast enhancement, were applied to preserve data authenticity. The infarcted area was manually traced and measured using ImageJ software (National Institutes of Health), and expressed as a percentage of the left ventricle relative to control.

### Echocardiography analysis of heart function

Mice were examined before surgery (baseline) and at 1, 4, and 8 weeks post-mI/R by transthoracic echocardiography under anesthesia (1–1.5% isoflurane) by blinded trained investigators (AL and YS). Images were acquired with a Vevo 3100 high-frequency ultrasound imaging system (VISUALSONICS, Inc, Toronto, Canada) fitted with a 30-MHz central frequency transducer and connected to an integrated rail system III. All echocardiographic parameters were measured according to recommendations of the ESC Working Group on Myocardial Function in Adult Rodents (Zacchigna et al. 2021). LV end-diastolic and end-systolic dimensions were measured by parasternal short-axis M-mode echocardiography, and FS was calculated using the ultrasound machine program; LV internal diameters at end diastole (LVIDd) and end systole (LVIDs) and EF were calculated in 2D mode from the right parasternal four-chamber long-axis view, using the modified Simpson method. Pulsed Doppler parameters of mitral inflow (early peak diastolic velocity (E), late peak diastolic velocity (A), and the E/A ratio) were measured in apical four-chamber view. Pulsed Doppler tissue parameters measured at the mitral septal annulus were peak systolic velocity (S’), early peak diastolic velocity (E’), and late peak diastolic velocity (A’), and the E/E’ ratio was calculated. Isovolumic relaxation time (IVRT) was measured from synchronous left ventricular outflow tract flow and mitral flow and used to calculate the E/IVRT ratio.

### Protein sample preparation

Fresh LV tissue (~ 30 mg) from the infarct border zone of mice 24 h after mI/R or hiPSCMs (~ 8 × 10^6^cells) were washed in cold DPBS and processed for the isolation of subcellular protein fractions (see Supplementary Material) or gently homogenized in RIPA buffer (Thermo Fisher) supplemented with 100-fold diluted protease and phosphatase inhibitors to obtain the total protein fraction. Homogenates were centrifuged at 20,000 xg at 4ºC for 20 min, protein concentration in the supernatants was measured by the BCA method (Smith et al. 1985), and samples were aliquoted and stored at−80ºC.

### Western blotting

Proteins (35 μg) were denatured, separated by SDS-PAGE, and transferred to a polyvinylidene difluoride (PVDF) membrane (Merck Millipore, USA). Non-specific sites were blocked by incubating membranes with 5% (w/v) BSA in TBST (137 mM NaCl, 20 mM Tris, and 0.1% (v/v) Tween-20, pH 7.6), followed by overnight incubation at 4ºC with primary antibodies in blocking buffer. Membranes were then washed 5 times for 10 min each with TBST and incubated for 1 h at room temperature (RT) with the appropriate secondary antibody in blocking buffer. After a further 5 more 10 min washes in TBST, immunoreactive bands were detected by enhanced chemiluminescence (Amersham ECLTM Primer Western Blotting Detection Reagent.

(GE Healthcare) (RPN2232), using a ChemiDoc XRS + system with Image Lab software from Bio-Rad Laboratories. Band density was quantified with Gel-Pro Analyzer 3.1 software (Sigma). Molecular weight was determined by comparison with prestained protein markers (Precision Plus Protein™ Dual Color Standards, Bio-Rad 1,610,374). Target protein bands were identified based on their predicted molecular weights, as supported by previous data and antibody specifications. Antibody specificity was evaluated by testing multiple options, selecting those with high specificity and minimal off-target binding. For each experimental group, data were obtained from *n* = 7 mice per group for in vivo assays or *n* = 5 independent assays per group for in vitro experiments. Western blot analyses included two technical replicates per condition to ensure data consistency and reliability. The quantification presented in our study reflects the average values obtained from these independent biological samples, ensuring robust statistical analysis. Full-length, uncropped, and representative Western blot images from all studied samples, confirming the biological relevance of the selected bands, are provided in the supplementary materials (Figures S8–S11). Equal loading was monitored with antibodies to GAPDH (for cytosolic fractions) or H3 (nuclear fractions). Antibodies and their dilutions, sources, and references are summarized in Table 1.

**Table 1:** Antibodies and dilutions

		**Protein**	**Provider**	**Code**	**Dilution**
**Antibodies**	1º	Bax	Cell Signaling	#2772	1:4000
		Bcl2	Cell Signaling	#3498	1:2000
		NRF2 (D1Z9 C)	Cell Signaling	#12,721	1:1000
		Phopho- PLN	Badrilla	A010-12 AP	1:3000
		PLN	Badrilla	A010-14	1:10,000
		DWORF	Mybiosource	MBS5400388	1:5000
		SERCA 2a	Cell Signaling	#4388	1:1000
		GAPDH	SIGMA	G9545-100UL	1:5000
		Keap1	Cell Signaling	#8047	1:5000
		P62	Cell Signaling	#5114	1:5000
		LC3 A/B	Cell Signaling	#4108	1:2000
		HO-1	Cell Signaling	#43,966	1:2000
		Histone H3	Cell Signaling	#9715	1:1000
	2º	ECL Mouse IgG, HRP-linked whole Ab	Promega	W402B	1:10,000
		ECL Rabbit IgG, HRP-linked whole Ab	Promega	W401B	1:5000

### In vitro knockdown

hiPSCMs were transfected with specific siRNAs targeting *NRF2* (Accell Human NFE2L2 siRNA, DHARMACON) or *DWORF* (SIRGT66230 WQ-2OMe, CREATIVE BIOLABS) using Lipofectamine™ MessengerMAX™ Transfection reagent (Thermo Fisher). Cells were seeded 2 days before transfection in 6-well plates with 2 mL STEMdiff Cardiomyocyte Support Medium (STEMCELL Technologies) at 60% confluence. Stock transfection mixes were prepared according to the manufacturer’s instructions. In brief, 9 μL Lipofectamine reagent was diluted in 141 μL Opti-MEM I Medium (Invitrogen) and incubated for 5 min at RT. In another tube, siRNAs or a scrambled control RNA oligonucleotide were diluted with Opti-MEM to a final concentration of 25 nM. The mixes were then combined and incubated for 30 min at RT to allow the formation of RNA–lipid complexes. For transfections, the STEMdiff Cardiomyocyte Support Medium was removed and replaced with 1.75 mL fresh medium and 250 μL of the appropriate transfection mix. The cells were incubated at 37 °C for 48 h, after which they were washed twice with DPBS at 37 °C before ischemia. Transfection efficiency was determined and is shown in Figure S3.

### RNA extraction and quantitative RT-PCR

Fresh samples of LV infarct border zone (~ 30 mg) from mice at the indicated time after mI/R or hiPSCMs (2 × 10^6^ cells) were washed with cold DPBS. Cells were pelleted by centrifugation at 480 × g for 10 min at 4 °C, whereas tissue samples were placed in a pre-chilled glass Petri dish in an ice bath and chopped with sharp scissors. RNA was extracted with the RNeasy Mini Kit (QIAGEN), and cDNA was prepared with the iScript cDNA Synthesis Kit (Bio-Rad). Quantitative real time polymerase chain reaction (RT-qPCR) was performed with the TB Green Premix Ex Taq II (Tli RNase H Plus) Master Mix (Takara Bio).

Glyceraldehyde 3-phosphate dehydrogenase (*GAPDH*) was used as the housekeeping control gene. Primers were obtained from Merck, and sequences are listed in Table 2.

**Table 2:** Primer sequences used for quantitative real-time PCR analysis

	**Primer**	**Forward (5'−3')**	**Reverse (5'−3')**
**Human**	*GAPDH*	TCAACGACCACTTTGTCAAGCTCA	GCTGGTGGTCCAGGGGTCTTACT
	*NANOG*	CATGAGTGTGGATCCAGCTTG	CCTGAATAAGCAGATCCATGG
	*NKX 2–5*	CTACGGTTATAACGCCTACCC	CGAAGTTCACGAAGTTGTTGTT
	*NRF2*	GCGCAGACATTCCCGTTTGT	GCTCTCGATGTGACCGGGAA
	*OCT4*	TCTTTCCACCAGGCCCCCGGCTC	TGCGGGCGGACATGGGGAGATCC
	*cTnT*	GGCAGCGGAAGAGGATGCTGAA	GAGGCACCAAGTTGGGCATGAACGA
	*DWORF*	TTCTTCTCCTGGTTGGATGG	TCTTCTAAATGGTGTCAGATTGAAGT
**Mouse**	*Nrf2*	TGCTCGGACTAGCCATTGCC	GTCTTGCCTCCAAAGGATGTCA

### Co-immunoprecipitation

hiPSCM extracts (8 × 10^6^ cells/extract) were collected and incubated overnight with an antibody to PLN (Badrilla, A010-14, 1:100), SERCA (Cell Signaling, 4388, 1:100) or DWORF (Mybiosource, MBS5400388, 1:500) at 4 °C with gentle shaking. To the incubation was added 20 μL of protein A–Sepharose slurry, followed by further incubation for 4 h at 4 °C with gentle shaking. The beads were then washed 3 times with immunoprecipitation (IP) buffer (20 mM HEPES pH 7.4, 0.5 mM EDTA, 150 mM NaCl, and 0.1% Triton X-100) to remove non-specifically bound proteins. In each wash, the beads were mixed gently with IP buffer and centrifuged for 10 min at 480 xg and 4 °C, and the supernatant was discarded. To elute antigen–antibody complexes, the beads were resuspended in 25 μL SDS gel-loading buffer and heated at 95 °C for 4 min. After this, samples were separated by SDS-PAGE at 30 mA constant current for 2 h. Immunocomplexes were analyzed by western blotting with primary antibodies to SERCA2a (Cell Signaling, 4388, 1:1000), PLN (Badrilla, A010-14, 1:10,000), or DWORF (Mybiosource, MBS5400388, 1:5000). IgG was used as a control.

### Isolation of microsomal fractions and determination of SERCA2a activity

Microsomal fractions enriched in sarcoplasmic reticulum (SR) vesicles were isolated as described in (Soler et al. 2015), with modifications. LV tissue samples (∼80 mg wet weight) obtained from the infarct border zone of mice 24 h after mI/R were homogenized in liquid nitrogen using a pestle and mortar in 10 mM NaCO_3_at 1:20 dilution, supplemented with protease inhibitors and phosphatase inhibitors. The homogenate was incubated on ice for 20 min and centrifuged at 5,900 xg for 10 min at 4 °C, and the supernatant was collected and centrifuged at 51,000 xg for 60 min at 4ºC. The resulting supernatant (cytosolic fraction) was discarded, and the pellet was resuspended in 0.6 mM KCl and resedimented at 100,000 xg for 40 min to obtain the microsomal fraction (SR vesicles). Protein concentration was measured by the BCA method (Smith et al. 1985), and samples were aliquoted and stored at − 80 ℃ until use.

SERCA2a activity was assayed in 96-well microplates using an enzyme-coupled, NADH-linked ATPase assay (Schaaf 2020) with modifications. Each well contained assay mix (50 mM MOPS pH 7.0, 100 mM KCl, 5 mM MgCl2, 1 mM EGTA, 0.2 mM NADH, 1 mM phosphoenol pyruvate, 10 IU/mL pyruvate kinase, 10 IU/mL lactate dehydrogenase, 1 μM A23187, 1 mM CaCl_2_ (free Ca^2+^, 10 µM), and 0.02 mg SR protein/mL). The assay was started by adding ATP to a final concentration of 5 mM (200 µL total assay volume/well), and absorbance was measured at 340 nm in a Clariostar microplate reader (BMG Labtech). As a control, SERCA pump activity was blocked with 100 nM thapsigargin (Lytton et al. 1991). Samples were tested in triplicate.

### Measurement of circulating sST2

Plasma samples isolated from mice 24 h after mI/R were assayed for sST2 levels, as previously described (Asensio-Lopez 2021)using an ELISA kit (Quantikine ELISA Mouse ST2/IL33R (MST200); R&D Systems, USA). Measurement at 24 h post-mI/R was based on a previous study showing an increase in circulating sST2 in acute-phase injury (Asensio-Lopez 2021). Reactions were terminated by addition of a stop solution, and absorbance was determined at 450 nm in a Clariostar microplate reader (BMG Labtech). Intra- and inter-assay precision in term of coefficient of variation were less than 10%.

### Statistical analysis

Data are reported as mean ± SEM. Statistical differences were evaluated by fitting linear models, with interactions determined by one-way ANOVA followed by post hoc testing with the Bonferroni correction. Differences were considered significant at *p* < 0.05.

## Results

### AEOL reduces reperfusion-induced succinate-driven mitochondrial ROS production

**Fig. 1:**
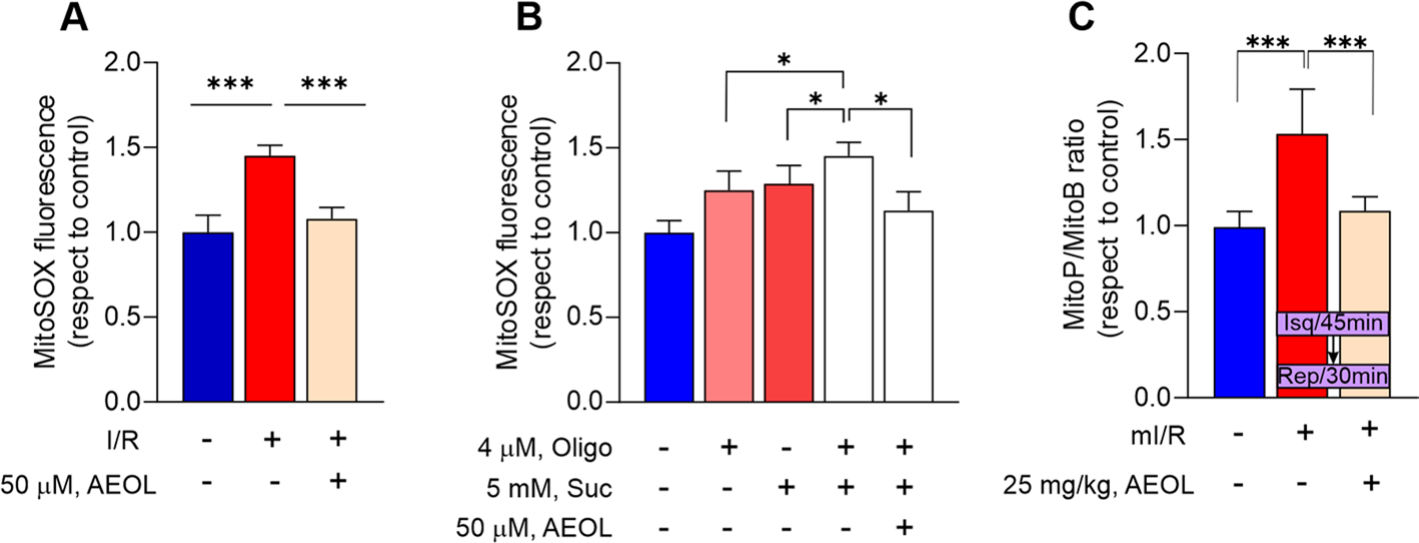
AEOL-10150 reduces ROS production upon reoxygenation/reperfusion. **A** ROS production assessed with the mitochondrial superoxide indicator MitoSOX Red in hiPSCMs subjected to simulated ischemia (1% O2, no glucose) for 45 min followed by 24 h reoxygenation (I/R model). Cells were treated with AEOL (50 µM) or vehicle (saline solution; 0.9% NaCl) at the onset of reoxygenation. **B** ROS production in hiPSCMs treated with dimethyl succinate (Suc) and oligomycin (Oligo) for 2 h. **C** Inhibitory effect of AEOL on ROS generation assessed 30 min after mI/R. Mice undergoing the mI/R procedure were treated with a subcutaneous injection of AEOL (25 mg/kg) or vehicle (DPBS) 15 min after reperfusion onset, and ROS were measured from the oxidation of MitoB injected 4 h before the procedure. Data are from *n* = 5 independent assays per group for in vitro procedures and *n* = 7 mice per group for in vivo procedures. Quantitative data are presented as mean ± standard error of the mean (SEM). ***p < 0.001; *p < 0.05, determined by one-way ANOVA followed by post hoc Bonferroni correction. BS, blood samples; CS, cardiac samples; I/R, ischemia–reperfusion; Oligo, oligomycin; Suc, succinate; wks, weeks. Other abbreviations are defined in the abbreviations list

We postulated that pharmacological scavenging of mitochondrial ROS (mtROS) by AEOL-10150 (hereafter AEOL) during mI/R would limit reperfusion-related injury, reduce cardiac remodeling, and protect against CHF. In the cellular model of simulated I/R, ischemia followed by reoxygenation triggered a significant increase in mtROS production by human iPS-derived cardiomyocytes (hiPSCMs) that was blocked by treatment with AEOL at the onset of reoxygenation (*p* < 0.001) (Fig. 1A). Since mtROS generation during reperfusion is driven by succinate (Tabata Fukushima et al. 2024); in separate experiments we treated hiPSCMs with cell-permeable dimethyl succinate and the ATP synthase inhibitor oligomycin, to mimic reperfusion-mediated mtROS production. In the context of the high ∆Ψm induced by ATP synthase inhibition, succinate fueled extensive mtROS generation, which was reduced by AEOL (Fig.1B). To investigate the effect of AEOL on cardiac mtROS production during mI/R in vivo, we used a model of left anterior descending coronary artery ligation and reperfusion (Figure S2; experimental groups −1a and −1b) in mice previously injected with the mitochondria-targeted ROS probe MitoB.

Treatment with AEOL 15 min after reperfusion onset prevented mtROS production during the early injury phase (30 min; Fig. 1D), resulting in attenuation of oxidative DNA damage (Figure S4 A). Similar results were obtained when mtROS levels were measured at 24 h post-reperfusion (Figure S4B).

### Short-term post-reperfusion AEOL therapy attenuates acute I/R injury and prevents adverse cardiac remodeling

**Fig. 2:**
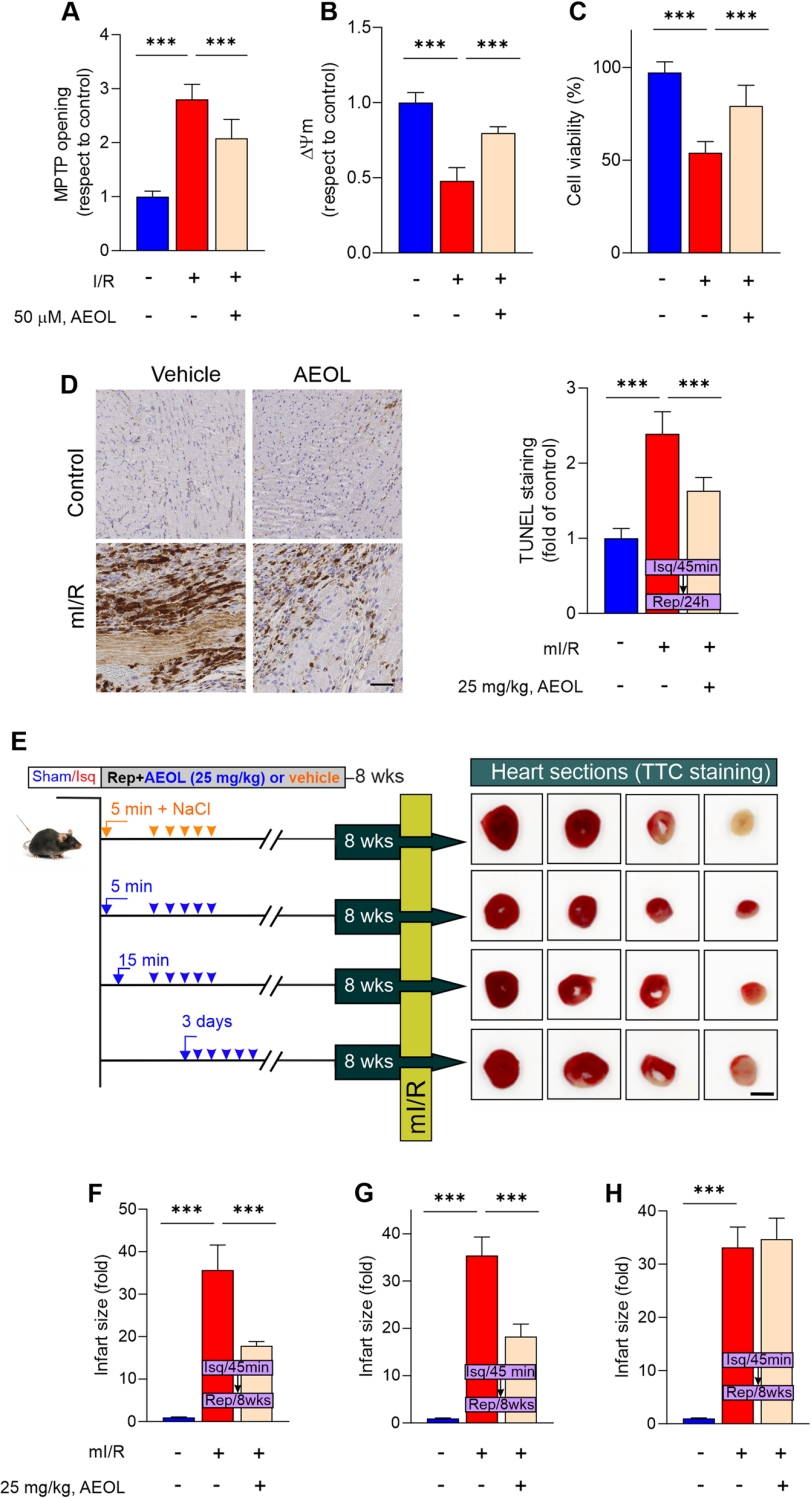
AEOL treatment reduces in the reactive oxygen species (ROS) surge triggered by reperfusion and protects against acute ischemia–reperfusion (I/R) injury. **A**-**C**, hiPSCMs subjected to ischemia(1% O2) for 45 min followed by 24 h reoxygenation (I/R model) were treated with AEOL (50 µM) and assayed for (**A**) mitochondrial permeability transition pore opening, **B** mitochondrial potential (Δψm), and (**C**) cell viability. **D**, Representative images and quantification of TUNEL staining in LV sections from mice subjected to mI/R and recovered for 24 h. Mice were treated as indicated with 25 mg/kg AEOL or vehicle (0.9% NaCl) 15 min after starting reperfusion. Scale bar, 50 µm. **E** Experimental design for determination of myocardial infarct size in mice subjected to mI/R and recovered for 8 weeks, with serial daily treatment with AEOL or vehicle (0.9% NaCl) starting 5 min, 15 min, or 3 days after reperfusion onset. Representative photographs show TTC staining 8 weeks post-reperfusion. **F**–**H** Effect of AEOL administration 5 min (F), 15 min (**G**), or 3 days (**H**) after reperfusion onset on infarct size determined at 8-weeks post-mI/R. Quantifications were obtained from *n* = 5 independent assays per group for in vitro procedures and *n* = 7 mice per group for in vivo assays. Quantitative data are presented as mean ± SEM. ***p < 0.001 determined by one-way ANOVA followed by post hoc Bonferroni correction. TTC, 2,3,5-triphenyltetrazolium chloride. Other abbreviations are defined in the abbreviations list

Mitochondrial function was impaired in the hiPSCM I/R model, as indicated by MPTP opening and collapse of the ∆Ψm (Fig. 2A-B). This resulted in activation of the mitochondrial apoptotic pathway, evidenced by upregulation of Bax protein expression and downregulation of Bcl2 (Figure S5 A). These alterations were prevented by AEOL treatment during the reoxygenation/recovery phase, which also attenuated cell death (Fig. 2C), reduced lactate accumulation, and restored hiPSCM ATP content (Figure S5B-C).

AEOL also decreased myocardial apoptosis in infarcted mice (Fig. 2D). Administration of AEOL either 5 min (Fig. 2E and F) or 15 min after reperfusion onset (Fig. 2E and 2G) and maintained through daily injections for the first 6 days after mI/R was able to reduce infarct size at 8 weeks. However, no cardioprotective effect was observed when AEOL administration was initiated later, starting at 3 days post-mI/R (Fig. 2E and H).

**Fig. 3:**
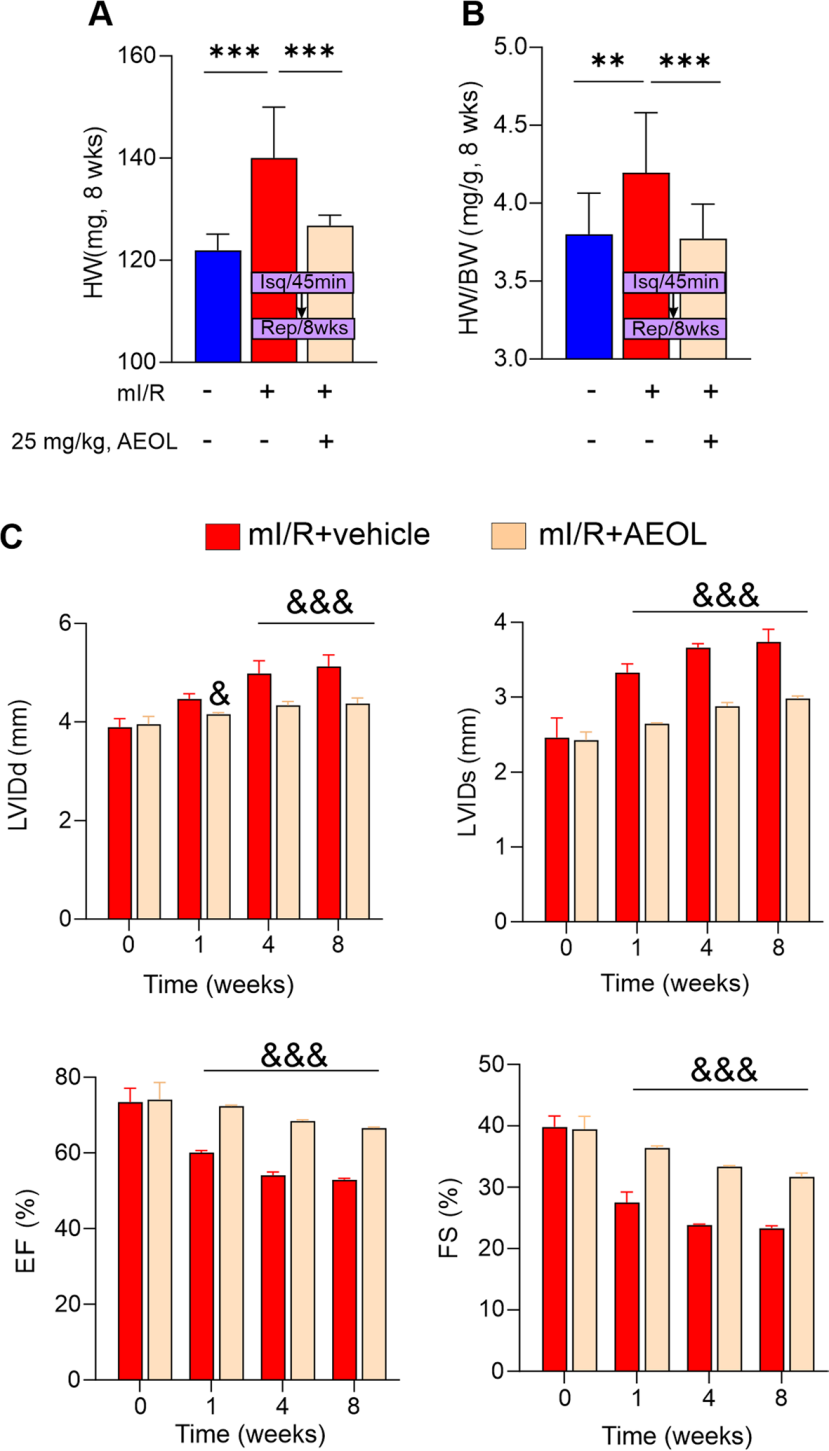
AEOL treatment in acute-phase mI/R injury improves cardiac function. **A**, **B** Heart weight (HW) and HW/body-weight (BW) ratio in mice subjected to mI/R, treated with AEOL or vehicle (0.9% NaCl) 15 min after reperfusion onset, and recovered for 8 weeks. **C** Evolution of echocardiography measures of cardiac function. Functional benefits of short-term AEOL treatment vs vehicle after mI/R include smaller LV internal diameters at end diastole (LVIDd) and end systole (LVIDs) and better preserved % ejection fraction (% EF) and % fractional shortening (% FS) (full echocardiography data are presented in Table 3). Data were obtained from *n* = 7 mice/group, and are presented as mean ± SEM. ***p < 0.001; **p < 0.01 or & *p* < 0.01; &&& *p* < 0.001 mI/R + AEOL vs mI/R + vehicle, determined by one-way ANOVA followed by post hoc Bonferroni correction. BW, body weight; HW, heart weight. Other abbreviations are defined in the abbreviations list

When AEOL therapy was initiated in the acute-phase (15 min post-mI/R), mice developed significantly less cardiac hypertrophy at 8 weeks than mice treated with vehicle (*p* < 0.01) (Fig. 3A-B). Taken together, these data suggest that short-term AEOL therapy after mI/R is protective and produces beneficial long-term outcomes. To test whether these differences in post-mI/R cardiac enlargement correlate with changes in cardiac structure and function, we examined mice by echocardiography. Acute-phase treatment with AEOL, with 6 daily injections starting at 15 min after mI/R, significantly reduced the severity of adverse remodeling detected after 8 weeks (*p* < 0.001) (Fig. 3B, Table 3).

**Table 3:** Echocardiographic parameters for cardiac functions

		**EXPERIMENTAL GROUPS**					
		**Sham (***n*** = 10)**	**C57BL6/mI/R + vehicle (***n*** = 14)**	**C57BL6/mI/R + AEOL (***n*** = 14)**	***Nrf2* KO/mI/R + Vehicle (***n*** = 8)**	***Nrf2* KO/mI/R + AEOL (***n*** = 10)**	***Nrf2*-KO/mI/R + AEOL + DWORF (*n* = 7)**
		**Post-mI/R (1 weeks)**					
**VARIABLES**	HR (bpm)	423.80 ± 10.46	415.90 ± 11.82	440.50 ± 9.38	419 ± 10.28	402.43 ± 9.94	441.29 ± 21.26
	LVEdD (mm)	3.97 ± 0.04	4.51 ± 0.02*	4.16 ± 0.01&	4.61 ± 0.13	4.52 ± 0.01	4.14 ± 0.00#
	LVEsD (mm)	2.50 ± 0.07	3.33 ± 0.04*	2.65 ± 0.02&	3.55 ± 0.12	3.24 ± 0.01	2.63 ± 0.01#
	FS (%)	40.49 ± 0.38	27.53 ± 0.54*	36.42 ± 0.10&	25.95 ± 0.99	28.24 ± 0.06	36.36 ± 0.05#
	FE (%)	75.66 ± 0.88	60.12 ± 0.16*	72.36 ± 0.08&	59.41 ± 0.53	60.66 ± 0.12	71.72 ± 0.34#
	CO (ml/min)	15.37 ± 0.36	10.62 ± 0.29*	12.54 ± 0.17&	10.15 ± 0.39	10.41 ± 0.23	12.78 ± 0.27#
	S (mm/s)	20.76 ± 0.93	17.68 ± 0.67*	20.26 ± 1.07&	16.65 ± 1.03	17.61 ± 0.76	22.06 ± 1.21#
	E/A	1.47 ± 0.03	1.96 ± 0.09*	1.46 ± 0.04&	1.95 ± 0.09	1.97 ± 0.10	1.52 ± 0.05#
	E/E´	30.98 ± 1.76	41.13 ± 1.16*	33.64 ± 2.87&	39.95 ± 1.60	40.95 ± 1.10	33.09 ± 1.17#
	E/IVRT	37.26 ± 0.97	20.35 ± 0.73*	36.53 ± 1.07&	22.56 ± 1.69	20.77 ± 0.82	38.55 ± 0.95#
		Post-mI/R (4 weeks)					
	HR (bpm)	412.00 ± 11.37	395.65 ± 43.83	455.10 ± 2.44	446.29 ± 9.60	432.86 ± 11.81	444.00 ± 9.32
	LVEdD (mm)	3.94 ± 0.08	4.83 ± 0.02*	4.34 ± 0.03&	4.89 ± 0.05	4.79 ± 0.01	4.37 ± 0.03#
	LVEsD (mm)	2.38 ± 0.03	3.66 ± 0.02*	2.88 ± 0.02&	3.68 ± 0.06	4.08 ± 0.43	2.90 ± 0.02#
	FS (%)	40.20 ± 0.35	23.84 ± 0.04*	33.34 ± 0.06&	23.59 ± 0.23	23.95 ± 0.02	33.49 ± 0.05#
	FE (%)	75.08 ± 0.86	54.10 ± 0.27*	68.43 ± 0.11&	52.72 ± 2.02	60.66 ± 0.45	63.85 ± 4.44#
	CO (ml/min)	15.38 ± 0.52	10.28 ± 0.64*	12.81 ± 0.32&	10.63 ± 0.67	11.87 ± 0.89	13.30 ± 0.51#
	S (mm/s)	20.54 ± 1.11	17.31 ± 0.62*	22.71 ± 0.60&	18.28 ± 0.43	17.16 ± 0.79	22.72 ± 0.69#
	E/A	1.39 ± 0.04	2.03 ± 0.13*	1.63 ± 0.09&	1.95 ± 0.13	2.01 ± 0.14	1.81 ± 0.09#
	E/E´	28.50 ± 1.41	43.84 ± 1.22*	32.26 ± 1.69&	38.04 ± 2.04	47.08 ± 5.84	37.54 ± 1.19#
	E/IVRT	39.98 ± 2.63	22.40 ± 1.08*	36.28 ± 1.11&	22.45 ± 1.41	23.90 ± 2.78	33.01 ± 1.133
		Post-mI/R (8 weeks)					
	HR (bpm)	427.50 ± 7.45	453.00 ± 18.30	423.70 ± 12.11	439 ± 6.84	440.29 ± 9.75	445.43 ± 7.74
	LVEdD (mm)	3.96 ± 0.04	4.98 ± 0.01*	4.37 ± 0.04&	5.02 ± 0.10	4.92 ± 0.01	4.38 ± 0.01#
	LVEsD (mm)	2.38 ± 0.04	3.74 ± 0.05*	2.98 ± 0.01&	3.59 ± 0.07	3.75 ± 0.02	2.98 ± 0.02#
	FS (%)	39.92 ± 0.24	23.33 ± 0.13*	31.70 ± 0.19&	24.01 ± 0.37	23.52 ± 0.05	32.12 ± 0.07#
	FE (%)	76.58 ± 0.38	52.88 ± 0.14*	66.57 ± 0.08&>	54.42 ± 1.16	52.93 ± 0.21	66.43 ± 0.15#
	CO (ml/min)	13.91 ± 0.33	9.61 ± 0.21*	12.74 ± 0.43&	9.88 ± 0.09	9.53 ± 0.17	12.70 ± 0.41#
	S (mm/s)	20.53 ± 0.93	17.42 ± 0.70*	21.73 ± 0.93&	16.85 ± 1.13	16.55 ± 0.52	23.99 ± 0.53#
	E/A	1.42 ± 0.05	1.90 ± 0.09*	1.53 ± 0.06&	1.88 ± 0.04	1.80 ± 0.03	1.72 ± 0.03#
	E/E´	29.38 ± 1.35	44.96 ± 2.76*	32.00 ± 3.31&	41.28 ± 4.57	36.73 ± 0.36	47.51 ± 2.21#
	E/IVRT	38.12 ± 2.35	21.30 ± 0.79*	32.35 ± 1.96&	19.49 ± 0.57	18.55 ± 1.03	34.89 ± + 0.48#

*HR* Heart rate, *LV* Left ventricle, *LVEdD* Left ventricular dimensions at end diastole, *LVEsD* Left ventricular dimensions at end systole, *FS* Fractional shortening, *EF* Ejection fraction, *CO* Cardiac output, *E* Early diastolic peak velocity of mitral valve flow, *E´* Early diastolic mitral annular velocity, *A* Late diastolic peak velocity of mitral valve flow, *IVRT* Isovolumic relaxation time. Data are expressed as mean ± SEM. *p < 0.05 vs. sham and mI/R groups; &*p* < 0.05 vs. mI/R + AEOL and mI/R + vehicle groups; #*p* < 0.05 vs. Nrf2 KO/mI/R + AEOL and Nrf2-KO/mI/R + AEOL + DWORF

Compared with vehicle-treated mice, AEOL-treated mice had smaller left ventricular (LV) internal diameters at end diastole (LVIDd) and end systole (LVIDs), accompanied by better % ejection fraction (% EF) and % fractional shortening (% FS). These functional and structural data thus demonstrate that AEOL reduces infarct expansion, improves outcomes, and limits progression to HF when therapy was initiated in the acute-phase (15 min post-mI/R).

### AEOL preserves the feed‐forward loop linking NRF2, Keap1, and p62 after mI/R

**Fig. 4:**
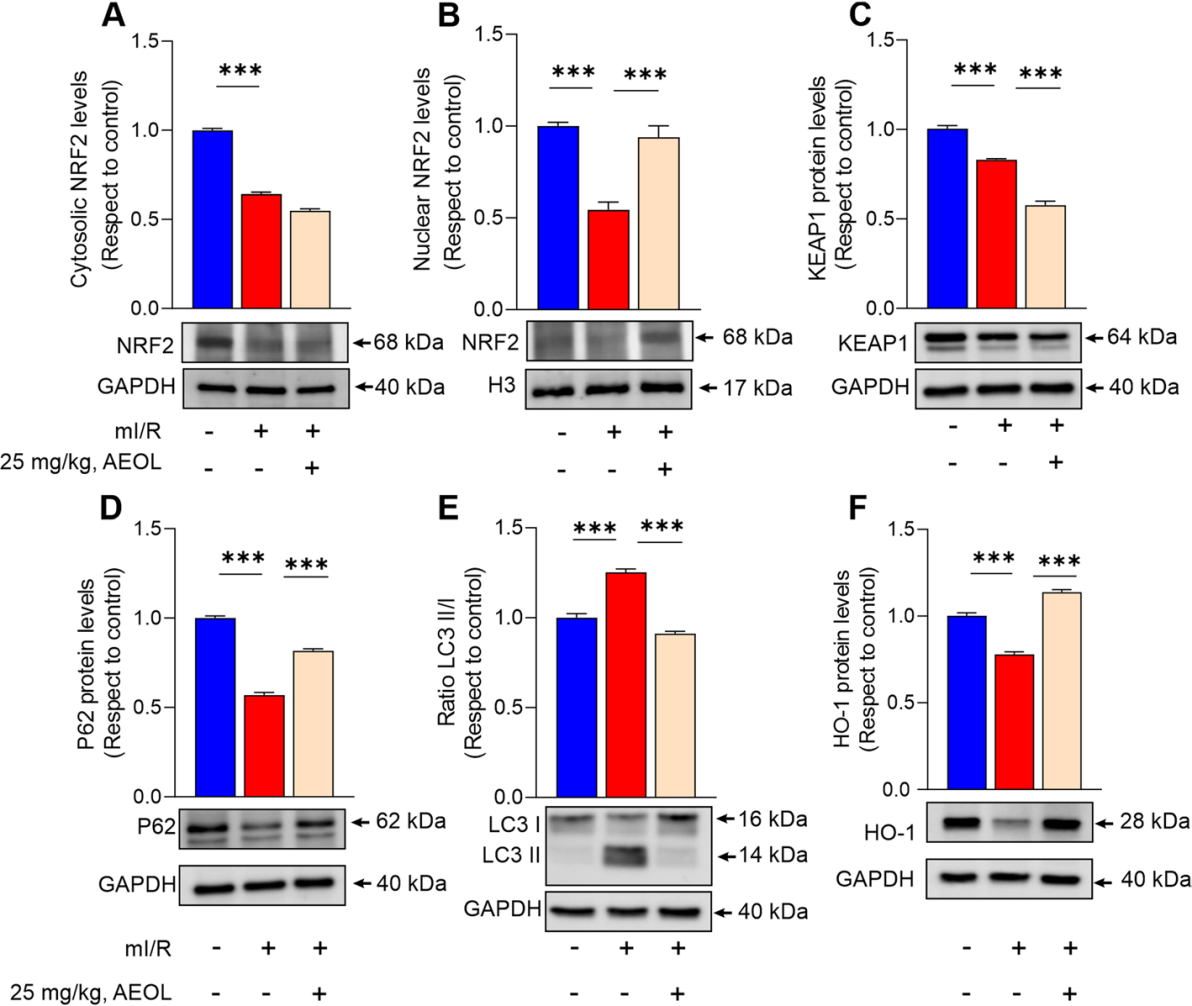
AEOL increases nuclear translocation of NRF2 and the transcriptional induction of downstream ARE-regulated genes in the infarct border zone. Mice subjected to mI/R were treated with AEOL or vehicle (0.9% NaCl) 15 min after reperfusion onset and sacrificed at 24 h post-mI/R. **A**,**B** Cytosolic and nuclear expression of NRF2 in the infarct border zone. **C** KEAP1 protein expression. **D** p62 protein expression levels. **E** LC3 II/LC3 I ratio. **F** HO-1 protein expression. Data are from *n* = 7 mice/group and are presented as mean ± SEM. ***p < 0.001; determined by one-way ANOVA followed by post hoc Bonferroni correction. GAPDH, glyceraldehyde phosphate dehydrogenase; H3, histone H3; HO-1, heme oxygenase 1; KEAP1, kelch-like ECH-associated protein 1; LC3, microtubule-associated protein 1 A/1B-light chain 3; NRF2, nuclear factor erythroid 2-related factor 2; p62, sequestosome 1. Other abbreviations are defined in the abbreviations list

To investigate the mechanism of the cardioprotective effect of early post-mI/R AEOL, we examined the effect on the p62–Keap1–NRF2 antioxidative signaling pathway. As early as 1-day post-mI/R, cytosolic and nuclear NRF2 localization in the myocardium was significantly decreased relative to sham-operated mice (Fig. 4A-B) (*p* < 0.001 in both cases).

This change was paralleled by decreases in the protein expression of Keap1 and p62 (Fig. 4C-D) (*p* < 0.001) and an increase in the cardiac LC3 II/I ratio (Fig. 4E) (*p* < 0.01). Furthermore, the disruption of the p62–Keap1–Nrf2 feed‐forward loop after mI/R resulted in direct impairment of antioxidant capacity, manifested as significantly reduced protein expression of HO‐1 (Fig. 4F) (*p* < 0.001) and the increased mtROS production presented in Fig. 1. AEOL treatment significantly increased p62 expression and normalized the LC3 II/I ratio (*p* < 0.001 in both cases). Cardiac nuclear NRF2 expression was significantly higher in AEOL-treated mI/R mice than in the vehicle-treated mI/R group (*p* < 0.001), although the Keap1 expression was further decreased by AEOL therapy (*p* < 0.01).

Close analysis of NRF2 activity and expression after mI/R revealed a surge in NRF2 activity and mRNA expression 15 min after reperfusion onset, followed by a decline to 24 h post-reperfusion (Figure S6). Treatment with 6 daily injections of AEOL starting at 15 min after mI/R sustained a significantly higher NRF2 activity than observed in control animals (*p* < 0.001), and this activation was maintained throughout the 8-week study period (Figure S6 A). These findings indicate that interventions targeting NRF2 activation after mI/R should be administered early after reperfusion.

### AEOL-induced NRF2 activation protects against I/R-induced cardiac injury

Given the ability of AEOL to preserve the feed‐forward loop linking NRF2, Keap1, and p62 during the acute injury phase, we next investigated the possible involvement of NRF2 in the cardioprotective effect of AEOL therapy.

**Fig. 5:**
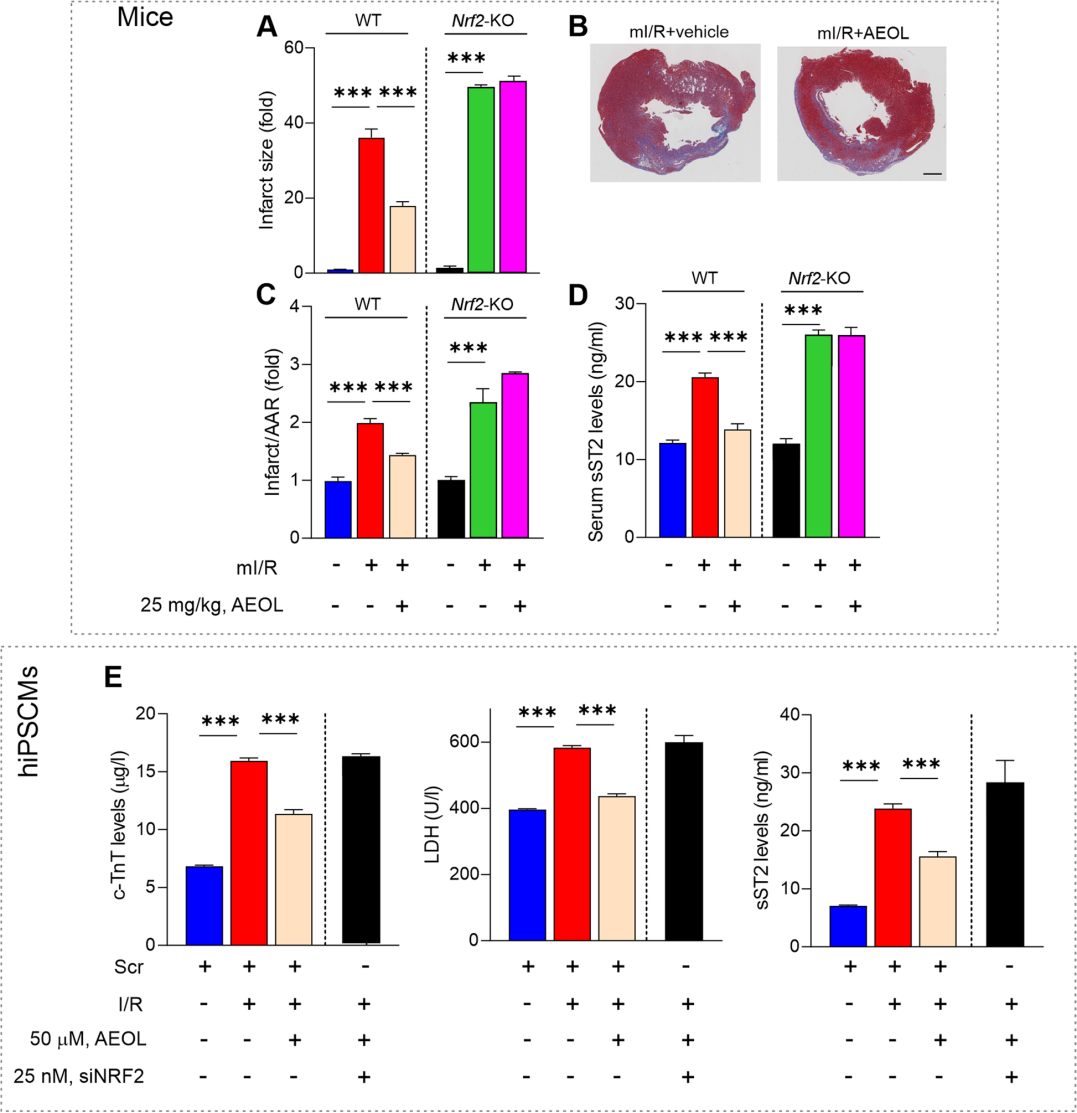
AEOL treatment ameliorates mI/R injury through NRF2. **A**-**D** Wild type and *Nrf2*-KO mice subjected to mI/R were treated with AEOL or vehicle (0.9% NaCl) 15 min after reperfusion onset and sacrificed at 8 weeks post-mI/R. **A** Infarct size calculated as the percentage of LV volume and expressed as fold of control. **B** Representative LV sections from wild type mice obtained 8 weeks post-mI/R. Collagen in the scar is blue and myocytes are red. Scale bar, 0.5 cm. **C** Area-at-risk (AAR) calculated as the percentage of LV volume and expressed as fold of control. **D** Plasma sST2 in mice at 24 h post-mI/R. **E** hiPSCM culture supernatant content of c-TnT, LDH, and sST2 at 24 h after simulated I/R. Quantifications were obtained from *n* = 7 mice per group for in vivo assays and *n* = 5 independent assays per group for in vitro procedures. Quantitative data are presented as mean ± SEM. ***p < 0.001 determined by one-way ANOVA followed by post hoc Bonferroni correction. AAR, area-at-risk; Scr, scramble; KO, knockout. Other abbreviations are defined in the abbreviations list

*Nrf2* knockout (KO) mice and (WT) littermate controls were treated or not with 6 daily injections of AEOL starting at 15 min after mI/R and assessed for mI/R injury after 8 weeks. Infarcts were larger in *Nrf2*-KO mice than in WT controls (*p* < 0.001), and whereas AEOL reduced infarct size in WT mice (*p* < 0.001), it had no effect in the *Nrf2*-KO mice. The same effect was evident when infarct size was expressed as a proportion of the area-at-the risk (AAR, Fig. 5B-C). Moreover, infarcted *Nrf2*-KO mice had much higher plasma levels of myocardial overload marker sST2 at 24 h post-mI/R than their WT counterparts, indicating more severe cardiac injury, and plasma sST2 was not reduced by AEOL therapy in the *Nrf2*-KO mice (Fig. 5D).

Comparable results were obtained in the hiPSCM I/R model. Specific siRNA silencing of human *NRF2* prevented AEOL-induced protection against increases in c-TnT, LDH, and sST2 after ischemia–reoxygenation (*p* < 0.001, in all cases) (Fig. 5E). These findings collectively suggest that AEOL exerts its cardioprotective action through modulation of NRF2.

### NRF2 improves cardiac contractility and prevents adverse cardiac remodeling through upregulation of DWORF protein expression

**Fig. 6:**
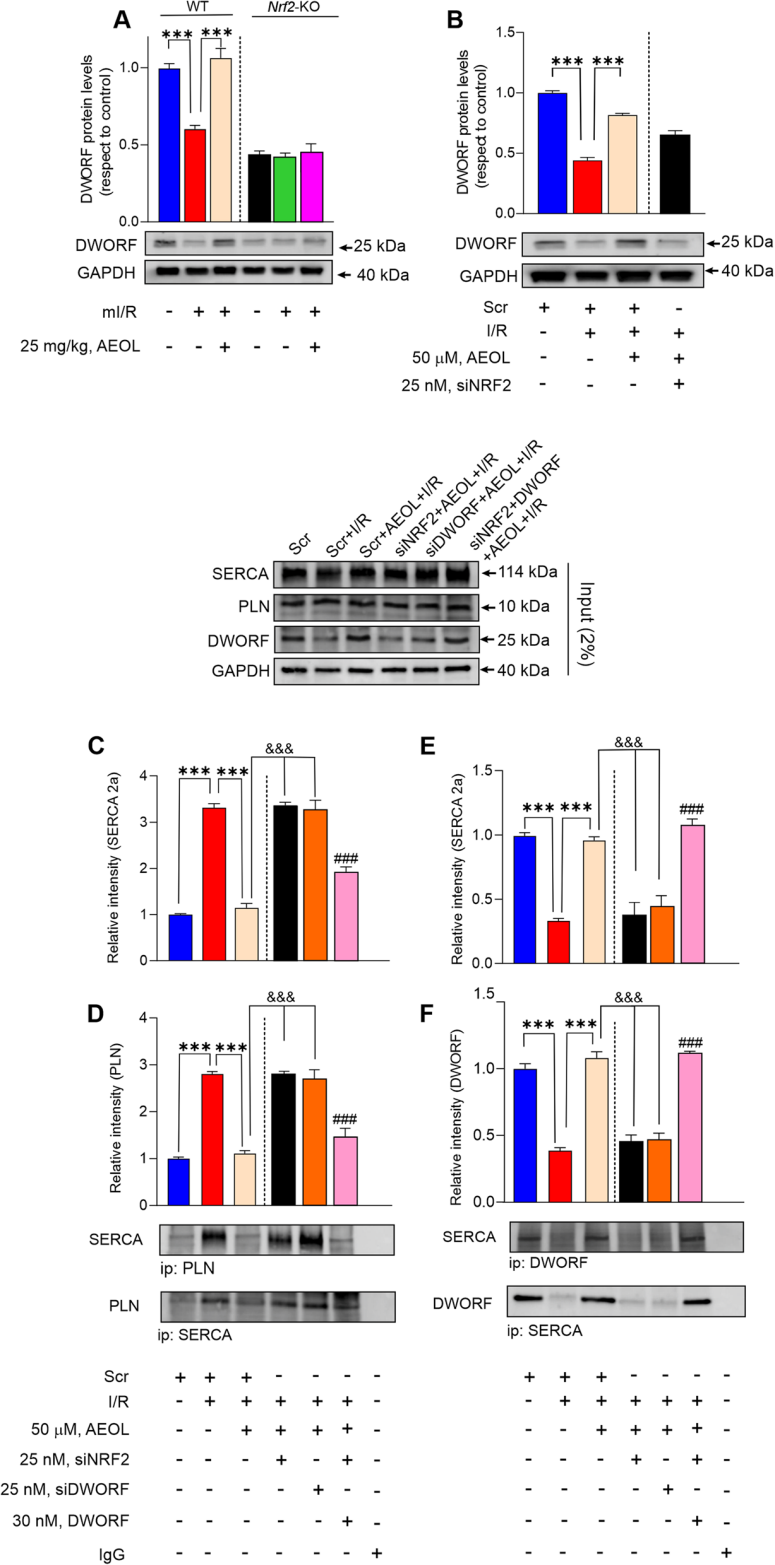
AEOL-induced DWORF upregulation displaces PLN from SERCA2a. **A** DWORF accumulation in the infarct border zone of mice 24 h after mI/R. Mice were treated with AEOL or vehicle (0.9% NaCl) 15 min after reperfusion onset. **B** DWORF accumulation in extracts of hiPSCMs subjected to the simulated I/R protocol (45 min ischemia followed by reoxygenation and maintenance for 24 h). Cells were transfected 48 h before simulated I/R with scrambled (Scr) or siNRF2 siRNA, as indicated, and treated with AEOL or vehicle (0.9% NaCl) at the onset of reoxygenation. **C**-**F** Co-immunoprecipitation analysis in hiPSCMs transfected as indicated with Scr, siNRF2, or siDWORF siRNA before simulated I/R. Cells were treated at the onset of reoxygenation with AEOL and DWORF, as indicated, and analyzed at 24 h after simulated I/R. **C**, **D** Co-immunoprecipitation and densitometry analysis of co-immunoprecipitated SERCA2a and PLN proteins. **E**, **F** Co-immunoprecipitation and densitometry analysis of co-immunoprecipitated SERCA2a and DWORF proteins. A representative input immunoblot is shown above panels C-F. Data were obtained from a sample size of *n* = 7 mice per group for in vivo assays and *n* = 5 independent assays per group for in vitro procedures and are presented as mean ± SEM. ***p < 0.001; ### *p* < 0.001 vs siNRF2 + AEOL + I/R, determined by one-way ANOVA followed by post hoc Bonferroni correction. Abbreviations are defined in the abbreviations list

In WT mice, mI/R reduced the protein expression of the SERCA2a activator DWORF measured in the infarct border zone 24 h after reperfusion onset (*p* < 0.001), and this effect was prevented by AEOL treatment at 15 min post-mI/R (*p* < 0.001). *Nrf2*-KO mice had lower baseline DWORF expression than WT mice, and this expression was unaffected by the mI/R procedure with or without post-reperfusion AEOL treatment (*p* < 0.001 in all cases) (Fig. 6A). Similar results were obtained in the simulated I/R model in hiPSCMs (Fig. 6B), with AEOL ameliorating the ischemia–reoxygenation-induced decrease in DWORF expression (*p* < 0.001). As in the mouse mI/R model, suppression of NRF2 expression reduced DWORF expression and blocked the effect of AEOL (*p* < 0.001).

We next tested the effect of NRF2-induced DWORF overexpression on the phosphorylation state of PLN, its interaction with SERCA2a, and cardiac contractility. Phosphorylation of PLN at Ser^16^ was decreased at 24 h post-mI/R in WT mice (*p* < 0.001), and this change was not prevented by AEOL treatment (Figure S7 A). The same pattern was observed in *Nrf2*-KO mice (*p* < 0.001), indicating that PLN phosphorylation is insensitive to NRF2 and the presence of AEOL (Figure S7 A). Comparable results were obtained in hiPSCMs, with simulated I/R injury decreasing PLN phosphorylation, AEOL treatment during reoxygenation unable to prevent it, and *Nrf2* knockdown having no effect (Figure S7B).

Co-immunoprecipitation analysis in hiPSCMs revealed that simulated I/R induced the formation of SERCA2a–PLN complexes, that AEOL treatment prevented the accumulation of these complexes, and that the effect of AEOL was annulled by silencing of endogenous NRF2 or DWORF protein expression (*p* < 0.001 in all cases) (Fig. 6C-D). Addition of exogenous DWORF protein to hiPSCMs with suppressed NRF2 expression restored the AEOL sensitivity of SERCA–PLN interaction (*p* < 0.001). In contrast, interaction between SERCA2a and DWORF was decreased after simulated I/R, and this decrease was prevented by AEOL treatment during reoxygenation (*p* < 0.001, in all cases) (Fig. 6E-F). The effect of AEOL was blocked by silencing of NRF2 or DWORF (*p* < 0.001), and addition of exogenous DWORF protein reverted the effect of siNRF2 (*p* < 0.001).

**Fig. 7:**
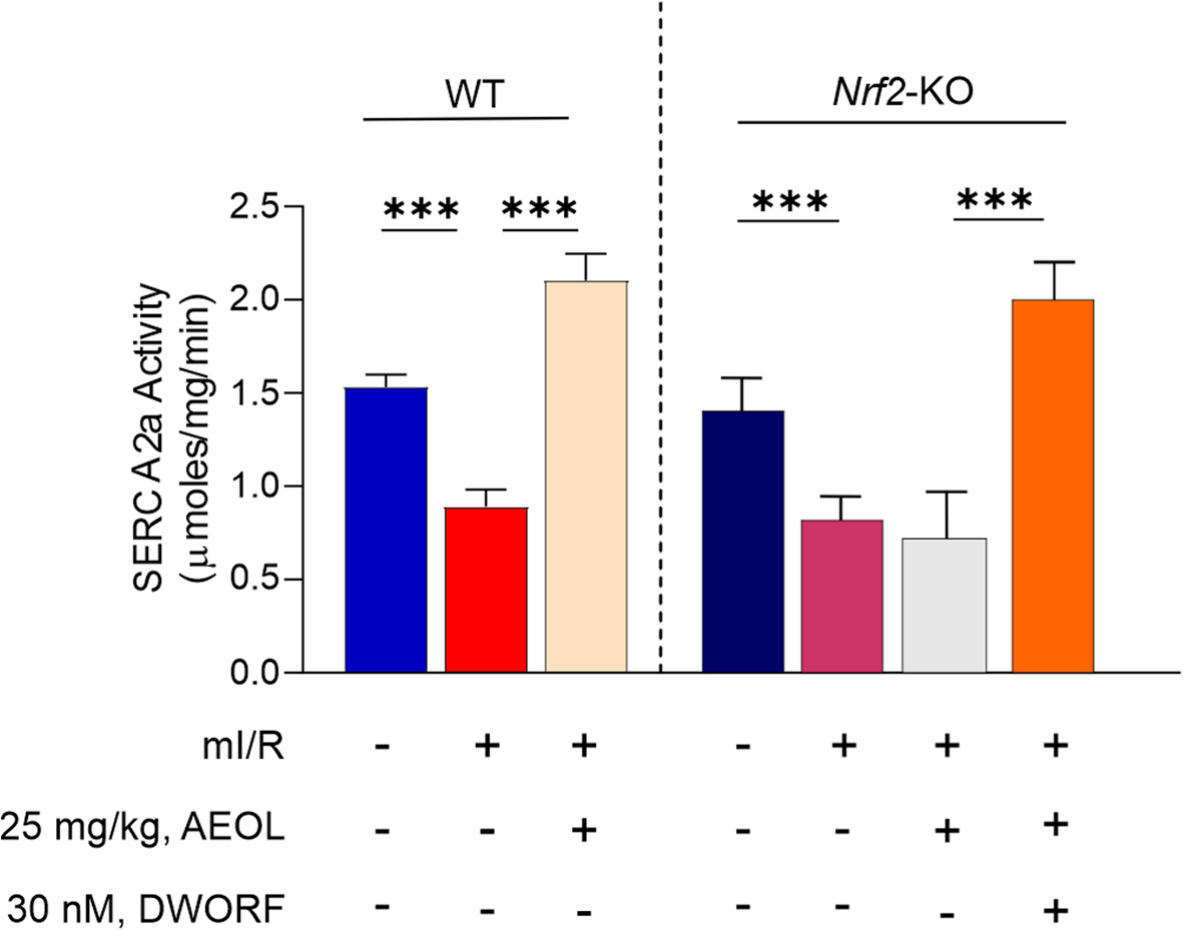
AEOL treatment enhances SERCA2a activity by upregulating DWORF expression. Maximal SERCA2a activity in the infarct border zone of wild type and Nrf2-KO mice subjected to mI/R, treated as indicated with AEOL and DWORF 15 min after reperfusion onset, and sacrificed at 24 h post-mI/R. Data were obtained from *n* = 7 mice/group and are presented as mean values ± SEM. ***p < 0.001, determined by one-way ANOVA followed by post hoc Bonferroni correction. Abbreviations are defined in the abbreviations list

Having established the ability of AEOL to regulate SERCA2a–PLN interaction through the cardiac micropeptide DWORF, we next investigated whether the DWORF–PLN interplay was able to regulate SERCA2a activity in the chronic phase after mI/R injury. SERCA2a activity was reduced at 24 h post-mI/R in both WT and *Nrf2*-KO mice (*p* < 0.001), and this effect was prevented by AEOL treatment at 15 min post-mI/R in WT mice (*p* < 0.001) but not in *Nrf2*-KO mice (Fig. 7).

Injection of DWORF in *Nrf2*-KO mice at the same time as AEOL overcame the blockade of AEOL-mediated protection of SERCA2a activity (*p* < 0.001) (Fig. 7) and improved cardiac function (Table 3).

## Discussion

This study demonstrates the potential applicability and therapeutic efficacy of AEOL in mitigating mI/R injury and preventing CHF. Our results provide evidence that AEOL-mediated acute activation of NRF2 protects against mI/R injury and establishes a causal link between AEOL-induced NRF2 activation and upregulated expression of the micropeptide DWORF, resulting in enhanced SERCA2a pump action and improved cardiac function. Our data also support the idea that DWORF serves as a direct activator of SERCA2a by displacing PLN from the SERCA2a complex at the onset of reperfusion.

For the present study, we sought to mimic the clinical scenario of myocardial remodeling, which is characterized by oxidative damage and a major pathophysiological impact of intracellular Ca^2+^ overload. The in vivo mouse model of 45 min occlusion of the left anterior descending coronary artery followed by reperfusion and recovery produced large infarct areas, occupying about 40% of the left ventricle and producing signs of CHF at 8 weeks post-reperfusion. Despite rapid interventional reperfusion strategies, a similar outcome can occur in patients with ST-segment elevation myocardial infarction, particularly those reperfused after prolonged ischemia or with low or no coronary reflow (Niccoli et al. 2009), conditions strongly associated with poor long-term outcomes.

Our research primarily focused on the role of NRF2 activation in mitigating mI/R injury, given its significance as a key defense mechanism against oxidative damage and mitochondrial dysfunction (Chen 2022;, Kasai 2020). Reductions in cardiac nuclear translocation of NRF2 and the expression of HO‐1 and p62 were accompanied by upregulated autophagy, increased apoptosis, and cardiac dysfunction after mI/R injury, effects that were prevented by AEOL treatment early after reperfusion onset. ROS accumulation increases significantly during the acute phase of cardiac reperfusion, triggering a reparative response associated with NRF2 activation (Wu et al. 2022). Unfortunately, this activation is transient, as evidenced by our finding of a substantial decline in*Nrf2*mRNA levels and activity just 24 h post-reperfusion. Enhancing NRF2 activity, including approaches promoting NRF2 nuclear translocation, is thus an attractive cardioprotective strategy with potential application in AMI (Wu et al. 2022; Zhu et al. 2019; –Orellana-Urzúa 2023). The deregulation of NRF2 signaling often precedes or exacerbates the progression of cardiovascular diseases (CVDs), and an increasing body of evidence supports the concept that boosting NRF2 levels, particularly during the early stages of cellular damage, reduces cell vulnerability and preserves cardiovascular system health. Consequently, investigating drugs that modulate NRF2 activity or its action pathways has become a crucial focus for the clinical management of CVDs. Researchers have actively pursued this strategy as an alternative therapy to achieve cardioprotection, and significant advances in understanding the NRF2 regulatory network in the coming years may lead to innovative therapeutic approaches for managing CVDs (Khan 2024). While previous studies examined AEOL’s potential to mitigate radiation-induced lung injury in large animal models (Cui et al. 2021;, Zhang 2018;, Garofalo et al. 2014;, Macvittie et al. 2017), alleviate anthracycline-induced cardiotoxicity (Kliment et al. 2009), and even reduce ischemic brain damage (Bowler et al. 2002), ours is the first study to explore the potential applicability and therapeutic efficacy of AEOL in mitigating cardiac mI/R injury and preventing CHF. Our results confirm that AEOL-induced cardioprotection during mI/R injury is largely dependent upon NRF2 activation and also define the most effective therapeutic intervention window, revealing that AEOL administered within 15 min of reperfusion onset, but not later, effectively attenuated mtROS production and averted oxidative DNA damage to cardiac tissue. Moreover, our findings provide compelling evidence that AEOL heightens mitochondrial performance and promotes cell viability, resulting in smaller infarcts and preserved myocardial function. These findings demonstrate the potential clinical utility of repurposing AEOL as a cardioprotective agent able to mitigate the deleterious effects of acute-phase reperfusion injury. Our study also firmly establishes the mechanistic relationship between the protective effect of AEOL and NRF2-pathway activation. The ability of AEOL to limit infarct expansion, adverse LV remodeling, and HF progression was abolished by*Nrf2*gene knockout; moreover, AEOL-induced NRF2 activation upregulated the expression of the micropeptide DWORF, a potent regulator of myocardial contractility via enhanced SERCA2a activity (Mbikou et al. 2020), achieved by displacing the SERCA-2a inhibitor PLN. Enhancement of SERCA-2a activity is concomitant with prominent Ca^2+^-loading of the sarcoplasmic reticulum and improved Ca^2+^ signal parameters, including an increase in the transient Ca^2+^ peak amplitude and a reduced cytosolic Ca^2+^influx decay time in each contraction–relaxation cycle (Makarewich 2018). One of the main causes of CHF is impaired myocardial Ca^2+^ cycling, which alters the heart's contractile function and structural remodeling. Ca^2+^ handling proteins, such the SERCA2a pump, play a major role in controlling the storage and release of Ca^2+^ in the sarcoplasmic reticulum of cardiomyocytes. The spatiotemporal patterns of intracellular Ca^2+^signaling, which regulate almost all cellular processes, including contraction, proliferation/hyperthrophic growth, and apoptosis, are mostly regulated by SERCA2a. One of the main characteristics of CHF is decreased SERCA2a expression and/or pump activity. Consequently, there is a growing interest in developing therapeutic approaches to target SERCA2a, directly or indirectly (Kho 2023;, Lipskaia et al. 2010). Enhancing or restoring SERCA2a activity has been shown in multiple studies to improve cardiac function and prevent the progression of HF in various animal models, providing strong evidence for the causal role of SERCA2a downregulation in CHF progression (Kawase et al. 2008; Byrne et al. 2008; Del 1999). Upregulated DWORF expression has been shown to restore Ca^2+^cycling and cardiac function in a mouse model of dilated cardiomyopathy (Makarewich 2018)and can promote cardioprotection in isolated rat hearts subjected to I/R (Mbikou et al. 2020); however, no previous study confirmed a cardioprotective effect of DWORF administered during cardiac reperfusion after an ischemic event in vivo or identified the factors controlling cardiac DWORF expression. Although our study did not focus on the detailed molecular mechanism, our data provide evidence of a causal relationship between NRF2 activation and cardiac DWORF upregulation after mI/R. Blocking NRF2 expression, through gene knockout in*Nrf2*-KO mice or siRNA gene silencing in hiPSCMs, abrogated AEOL-induced DWORF upregulation without affecting the phosphorylation status of PLN. This is an important observation given the blockade of SERCA2a activation in AEOL-treated *Nrf2*-KO mice after mI/R. This result can be explained by the ability of DWORF to activate SERCA2a by displacing PLN, regardless of its phosphorylation state (Fisher et al. 2021), as suggested by our data analysis. Treatment with DWORF in combination with AEOL in the acute reperfusion phase restored SERCA2a activation in*Nrf2*-KO mice and prevented subsequent development of systolic and diastolic dysfunction, mimicking the effect of AEOL treatment alone in wild-type mice.

## Conclusion

Our study demonstrates the therapeutic potential of AEOL as a modulator of the NRF2–DWORF pathway, ultimately leading to preserved cardiac contractility, reduced myocardial injury, and protection against HF development. These results have potential clinical implications, suggesting that AEOL, when administered early during reperfusion, may help mitigate reperfusion-induced injury and improve long-term cardiac outcomes in AMI patients. However, further research and clinical trials are needed to validate these preclinical findings and assess their translation into effective treatments for AMI patients.

### Study limitations

While our study establishes a causal link between AEOL-induced NRF2 activation and DWORF upregulation, the mechanism through which NRF2 induces DWORF expression remains to be elucidated. The upregulation of NRF2 after acute MI has cardiac protective effects via multiple underlying mechanisms, such as alleviating mitochondrial ROS and preventing cytochrome C release. Therefore, the protective effect observed in our study adds value to the current understanding of the cardioprotective mechanism and highlights the potential interest in new therapeutic targets beyond the DWORF-PLN-SERCA2a mechanism. Secondly, whereas NRF2 is known to be activated by heightened ROS levels, our analysis did not address the specific mechanism of AEOL-induced NRF2 activation. Further studies are warranted to explore this intriguing phenomenon. Moreover, although SERCA2a activity is assessed using enzyme-linked approaches, the functional impact of SERCA2a on SR Ca^2+^ load and diastolic intracellular Ca^2+^ level was not assessed. In the supplementary information, we provide representative Western blot images used in this study. Unfortunately, a critical failure in the storage system resulted in the loss of several replicates, preventing us from displaying all the complete images acquired. This limitation restricted our ability to present the full set of data for each experiment.

### Perspectives

#### Competency in medical knowledge

This study elucidates the significant role of AEOL-10150 (AEOL) as a novel NRF2 activator in mitigating myocardial ischemia–reperfusion (mI/R) injury. The findings demonstrate that AEOL reduces mitochondrial ROS production, decreases myocardial infarct size, and improves cardiac function through NRF2-mediated upregulation of DWORF, which enhances SERCA2a activity by disrupting the phospholamban-SERCA2a interaction. These results highlight the potential of AEOL as a therapeutic agent in treating mI/R injury, providing clinicians with novel insights into the molecular mechanisms involved in cardiac protection and advancing their medical knowledge in this domain.

#### Translational Outlook

Translating these findings from experimental models to clinical practice involves addressing several challenges. Key barriers include confirming the safety and efficacy of AEOL in large-scale clinical trials, optimizing dosage and administration protocols, and understanding potential side effects in diverse patient populations. Future research should focus on these aspects, as well as exploring the long-term benefits and any unforeseen impacts of chronic AEOL use. By overcoming these challenges, AEOL could be integrated into standard treatment regimens for myocardial infarction patients, enhancing recovery outcomes and reducing the progression to chronic heart failure.

## Supplementary Information

Supplementary Material 1.

## Data Availability

No datasets were generated or analysed during the current study.

## Abbreviations

∆Ψm: Mitochondrial membrane potential
AMI: Acute myocardial infarction
CHF: Chronic Heart Failure
DWORF: DWARF open reading frame
EC50: Half maximal effective concentration
HO-1: Heme oxygenase 1
Keap-1: Kelch-like ECH-associated protein 1
LC3: Microtubule-associated protein 1A/1B-light chain 3
MPTP: Mitochondrial permeability transition pore
NRF2: Nuclear factor erythroid 2-related factor 2
P62: Sequestosome 1
PLN: Phospholamban
ROS: Reactive oxygen species
SERCA2a: Sarcoplasmic reticulum Ca2 + -ATPase
mI/R: Myocardial ischemia–reperfusion
mtROS: Mitochondrial ROS
