# Correction: AEOL-induced NRF2 activation and DWORF overexpression mitigate myocardial I/R injury

**DOI:** 10.1186/s10020-026-01437-0

**Published:** 2026-03-04

**Authors:** Maria del Carmen Asensio-Lopez, Miriam Ruiz-Ballester, Silvia Pascual-Oliver, Francisco Jose Bastida-Nicolas, Yassine Sassi, Jose Javier Fuster, Domingo Pascual-Figal, María Josefa Fernandez del Palacio, Fernando Soler, Antonio Lax

**Affiliations:** 1 Centro Nacional de Investigaciones Cardiovasculares (CNIC), Madrid, Spain; 2 Centro Nacional de Investigaciones Cardiovasculares (CNIC), El Palmar, Murcia, Spain; 3 Instituto Murciano de Investigación Biosanitaria (IMIB) Pascual Parrilla and University of Murcia, Ctra. Madrid-Cartagena S/N, Murcia, Spain; 4 Fralin Biomedical Research Institute at Virginia Tech Carilion, Roanoke, Virginia USA; 5 CIBER en Enfermedades Cardiovasculares (CIBER-CV), Madrid, Spain; 6 Cardiology Department, Hospital Virgen de La Arrixaca, IMIB-Pascual Parrilla, University of Murcia, El Palmar, Murcia, Spain; 7 Veterinary Teaching Hospital, Veterinary Medicine and Surgery Department, University of Murcia, El Palmar, Murcia, Spain

**Correction: Molecular Medicine 31, 189 (2025)**

**https://doi.org/10.1186/s10020-025-01242-1**

In this article [1], the author Maria Josefa Fernandez del Palacio at affiliation “Veterinary Teaching Hospital, Veterinary Medicine and Surgery Department, University of Murcia, El Palmar, Murcia, Spain” is missing from the author list.

In addition to the above, the author Maria Josefa Fernandez del Palacio was included in the ‘‘Author contributions’’ section and the correct version of the ‘‘Author contributions’’ section is given below for reference.

Author contributions:

M.d.C.A.-L. and A.L. conceived the study, generated the experimental data, and wrote and edited the manuscript. A.L. is the guarantor of this work and, as such, has full access to all the data and takes responsibility for the integrity of the data and the accuracy of the analysis. M.d.C.A.-L. and A.L. confirm the authenticity of all the raw data. Echocardiography procedures, measurements, and interpretation were performed by M.J.F.-P., M.R.B., and S.P.-O. Data analysis was performed by M.J.F.-P., M.R.B., S.P.-O., and F.J.B.N. Y.S. contributed to writing the discussion. D.P.-F., F.S., and J.J.F. contributed to data analysis, discussion writing, and manuscript editing. All authors read and approved the final version of the manuscript and agree to be accountable for all aspects of the work.

The original article has been corrected.
