# Supplementary material for: AEOL-induced NRF2 activation and DWORF overexpression mitigate myocardial I/R injury
Source: Mol Med. 2025 May 15;31:189. doi: 10.1186/s10020-025-01242-1 (PMC12079873; doi:10.1186/s10020-025-01242-1)
Supplement: Supplementary file 1 — Supplementary Material 1. [file 10020_2025_1242_MOESM1_ESM.docx]

**Supplemental information**

AEOL-Induced NRF2 Activation and DWORF Overexpression Mitigate Myocardial I/R Injury.

María del Carmen Asensio-Lopez, Miriam Ruiz-Ballester, Silvia Pascual-Oliver, Francisco Jose Bastida-Nicolas, Yassine Sassi, Jose Javier Fuster, Domingo Pascual-Figal, Fernando Soler, and Antonio Lax.

**METHODS AND RESULTS EXPANDED**

**Methods**

*Subcellular fractionation*. Nuclear and cytosolic fractions were isolated from hiPSCMs and tissue samples as described previously[1], with modifications. Briefly, fresh mouse LV tissue (~ 30 mg) or harvested hiPSCMs (~3 × 10^6^ cells) were washed with cold DPBS supplemented with 100-fold diluted protease and phosphatase inhibitors. hiPSCMs were pelleted by centrifugation at 480 ×g for 10 min at 4°C, whereas mouse myocardium samples were placed in a pre-chilled glass Petri dish and minced on ice using sharp scissors. All samples were homogenized in 500 μl STM buffer, pH 7.4, (250 mM sucrose, 50 mM Tris-HCl, 5 mM MgCl_2_, 5 mM Na_3_VO_4_, 1% (v/v), and protease and phosphatase inhibitor cocktails) and homogenized for 1 min on ice using a tight-fitting Teflon pestle attached to a Potter S. homogenizer (Sartorius Stedim Biotech) set to 800 rpm. The homogenate was maintained on ice for 30 min, vortexed at maximum speed for 15 s, and then centrifuged at 800 ×g for 15 min at 4°C. The pellet was labeled P0 and kept on ice; the supernatant was labeled S0. The P0 pellet (containing nuclei and debris) was resuspended in 500 μl STM buffer, vortexed at maximum speed for 15 s, and then centrifuged at 500 ×g for 15 min at 4°C. The resulting nuclear pellet was labeled P1 and kept on ice, and the supernatant S1 (cell debris) was discarded. To increase purity, the P1 fraction was washed in 400 μl STM buffer, vortexed at maximum speed for 15 s, and centrifuged at 1,000 ×g for 15 min at 4°C. The washed pellet was labeled P2 (the S2 supernatant was discarded) and resuspended in 100 μl NET buffer (20 mM Hepes pH 7.9, 1.5 mM MgCl_2_, 0.5 M NaCl, 0.2 mM EDTA, 20% (v/v) glycerol, 1% (v/v) Triton-X-100, and protease and phosphatase inhibitors) using a pipette to triturate until homogeneity. The resuspended P2 pellet was vortexed at maximum speed for 15 s and incubated on ice for 30 min; the resulting fraction contained the nuclei, which were lysed by sonication (Soniprep 150, MSE) at high setting for 10–15 s with 30 s pauses while being kept on ice. The lysate was centrifuged at 9,000 ×g for 30 min at 4°C, and the resulting supernatant (S3) was the final nuclear fraction. Cytosolic fractions were extracted from S0 by centrifugation at 11,000 ×g for 10 min at 4°C. The resulting supernatant contained the cytosolic fraction. The protein content of each compartment was determined by BCA protein assay[2].

*NRF2 activity assay.* The activity of the transcription factor NRF2 was studied in a sandwich-based colorimetric assay (Q60795; RayBiotech, Inc). Nuclear extracts were obtained according to the manufacturer's guidelines from the infarct border zone of mice subjected to mI/R and recovered for 30min, 24h, 7d, 28d, or 8 weeks, with treatment with AEOL or vehicle (0.9% NaCl) by subcutaneous injection 15 min after reperfusion onset. The reaction was terminated by the addition of stop solution, and absorbance was determined at 450 nm in a Clariostar microplate reader (BMG labtech).

**Results**

1.-


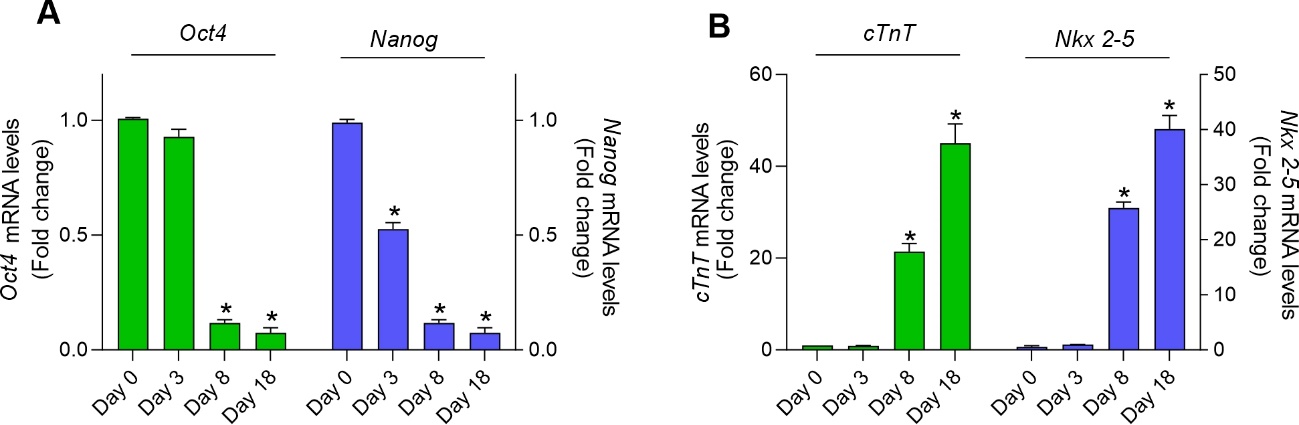


**Figure S1.**

*Relative gene expression of (a) pluripotency markers and (b) cardiac-specific markers in hiPSCMs.* The mean Ct values of triplicate measurements were normalized against the values for *GAPDH* for the same sample. Following normalization, the means of 3 independent assays were plotted, and the data are presented as mean ± SEM. * p<0.001 with respect to day 0, determined by one-way ANOVA followed by post hoc tests with Bonferroni correction.


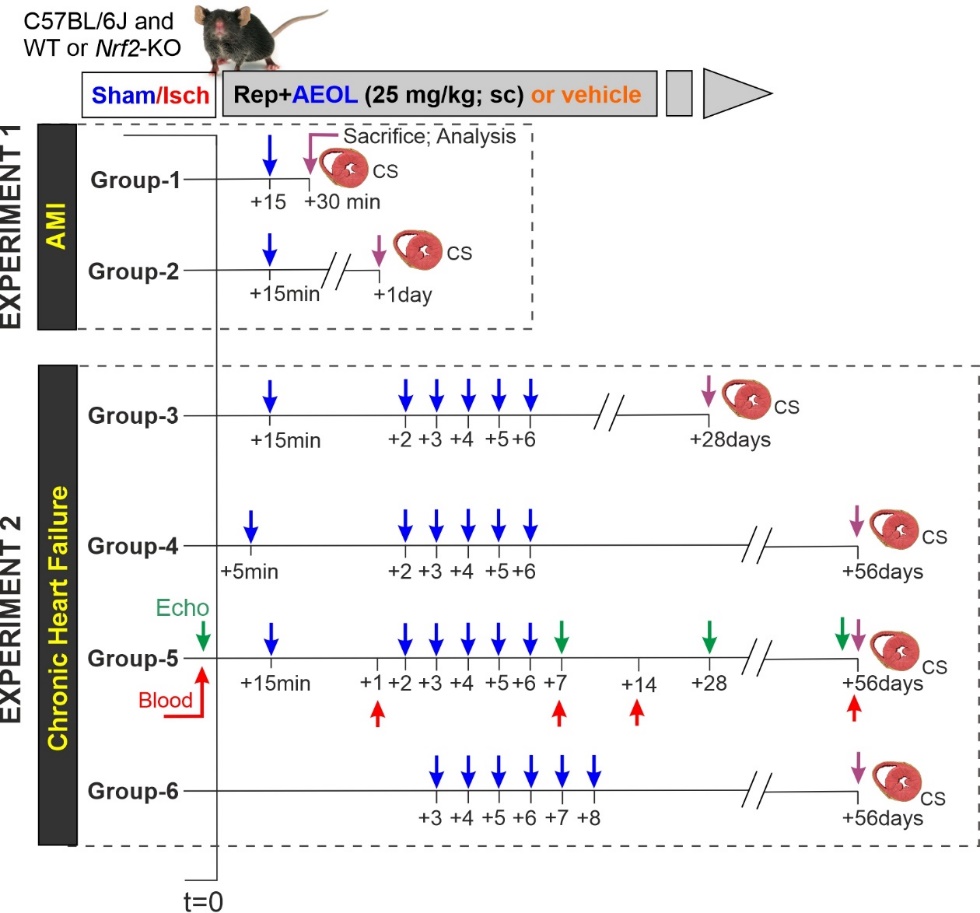
2.-

**Figure S2**. Experimental design for testing the effect of AEOL on the acute and chronic consequences of myocardial ischemia–reperfusion (mI/R) in mice (left anterior descending artery occlusion for 45 min followed by reperfusion [t=0] and recovery for the indicated time). Experiment 1. Mice receiving a single dose of AEOL or vehicle (0.9% NaCl) 15 min after reperfusion onset were allowed to recover for either 30 min or 24 h. Experiment 2. Mice received repeated daily injections of AEOL or vehicle for 5 days, starting at 5 min, 15 min, or 3 days after reperfusion onset, and were allowed to recover after mI/R for either 4 or 8 weeks. AEOL/vehicle treatment is denoted by the blue arrow, blood sample isolation by the red arrow, echocardiography analysis by the green arrow, and the endpoint experiment, involving sacrifice and biochemical analysis in isolated and processed cardiac samples (CS), is represented by the purple arrow. Other abbreviations are defined in the abbreviations list.

3.-


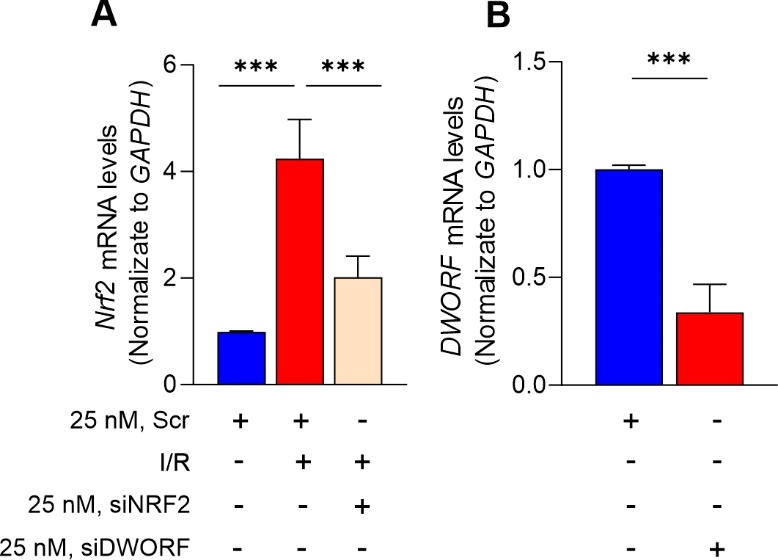


**Figure S3.** *SiNRF2 and siDWORF transfection efficiency.* hiPSCMs were preincubated with scramble siRNA (Scr) or with siRNA to NRF2 or DWORF. The mRNA expression of *NRF2* (A) and *DWORF* (*STRIT1*) (B) was analyzed by quantitative RT-PCR (normalized to *GAPDH*). Data are from n = 5 independent assays/group and are presented as mean ± SEM. ***p<0.001 determined by one-way ANOVA followed by post hoc Bonferroni correction. Scr, scramble. Other abbreviations are defined in the abbreviations list.


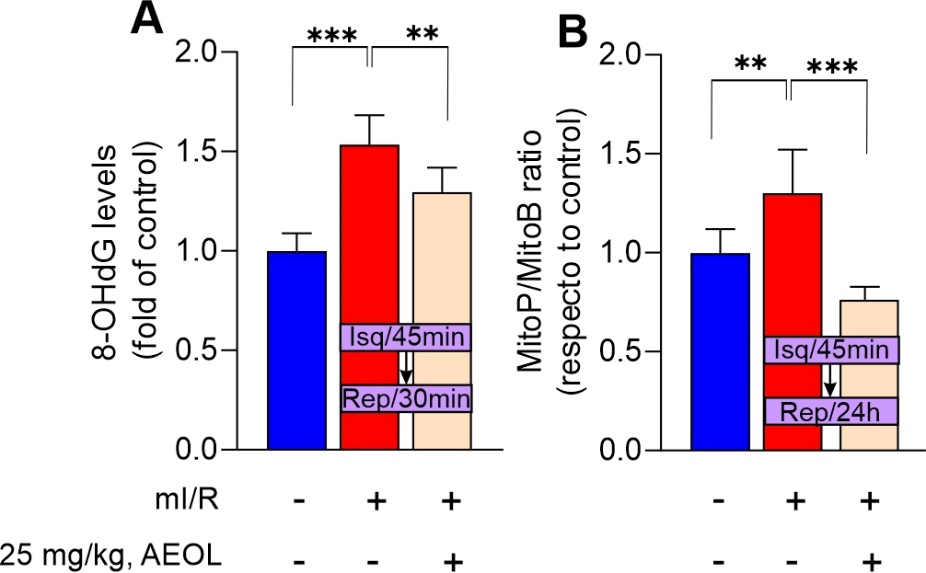
4.-

**Figure S4.** *AEOL treatment reduces cardiac oxidative damage upon reperfusion.* (A) Cardiac 8-hydroxy-2’-deoxyguanosine (8-OHdG) measured in the infarct border zone 30 min after mI/R. (B) mtROS measured in the infarct border zone 24 h after mI/R. Mice were treated as indicated with AEOL or vehicle (0.9% NaCl) 15 min after reperfusion onset. Data were obtained from n = 5 independent mice/group and are presented as mean ± SEM. ***p<0.001, **p<0.05 determined by one-way ANOVA followed by post hoc Bonferroni correction. Abbreviations are defined in the abbreviations list.

5.-

**
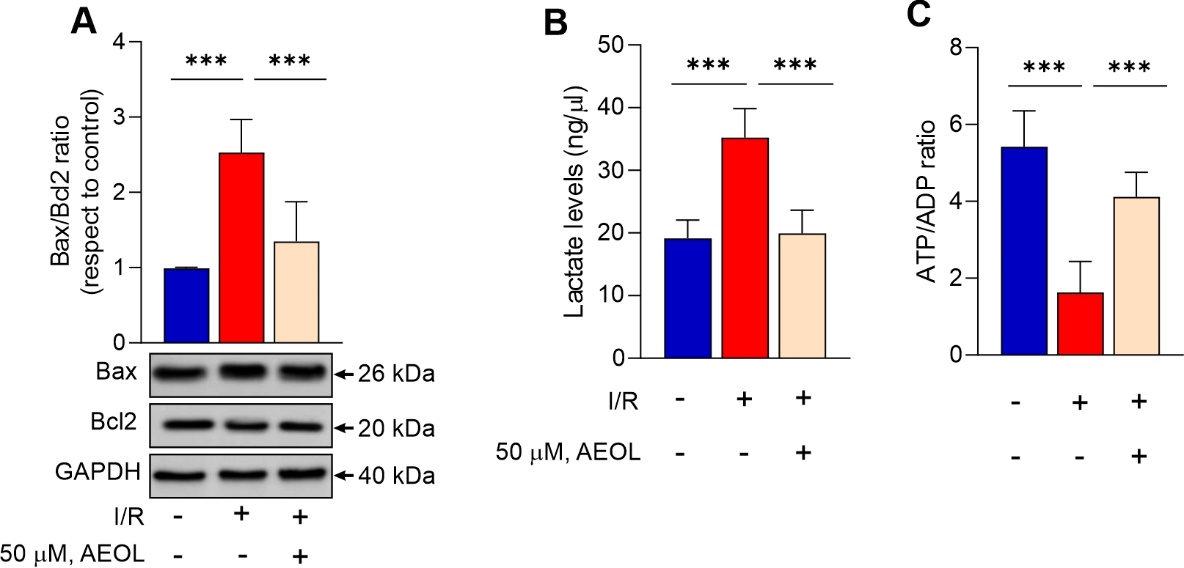
**

**Figure S5.** *AEOL treatment protects hiPSCMs against simulated I/R injury.* (A) Bax/Bcl2 ratio. (B) Lactate concentration in the culture supernatant. (C) ATP/ADP ratio. Data were obtained from n = 5 independent assays/group and are presented as mean ± SEM. ***p<0.001, determined by one-way ANOVA followed by post hoc Bonferroni correction. Abbreviations are defined in the abbreviations list.


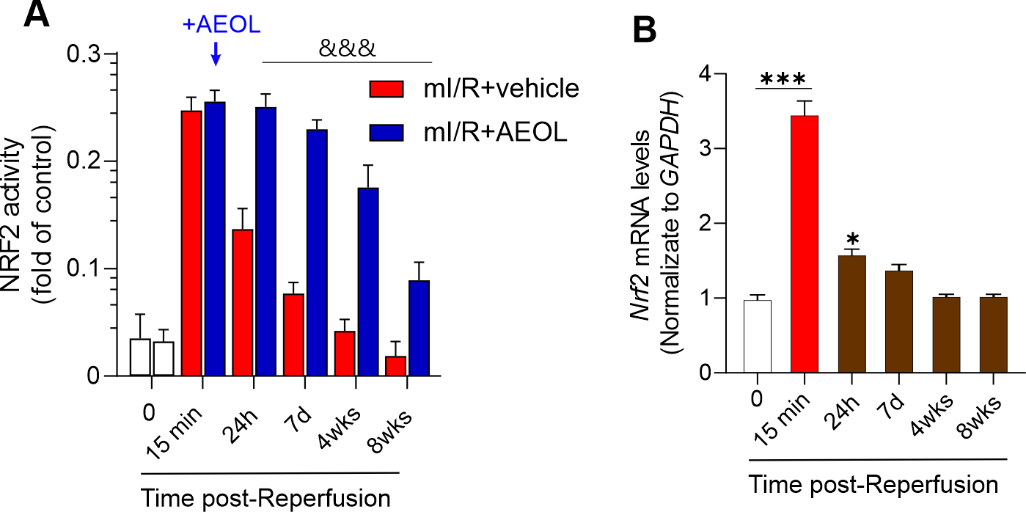
6.-

**Figure S6.** AEOL treatment in the acute-phase of mI/R injury maintains NRF2 activity by inducing *Nrf2* mRNA expression. (A) Evolution of NRF2 activity in the infarct border zone of mice subjected to mI/R and treated with AEOL or vehicle (DPBS) 15 min after reperfusion onset. (B) Quantitative reverse transcription polymerase chain reaction analysis of *Nrf2* mRNA (normalized to *GAPDH*) in the infarct border zone at the indicated times after mI/R. Data were obtained from n = 5 independent mice/group and are presented as mean ± SEM. &&& vs mI/R+vehicle; ***p<0.001, *p<0.05 vs sham operated, determined by one-way ANOVA followed by post hoc Bonferroni correction. Abbreviations are defined in the abbreviations list.

**
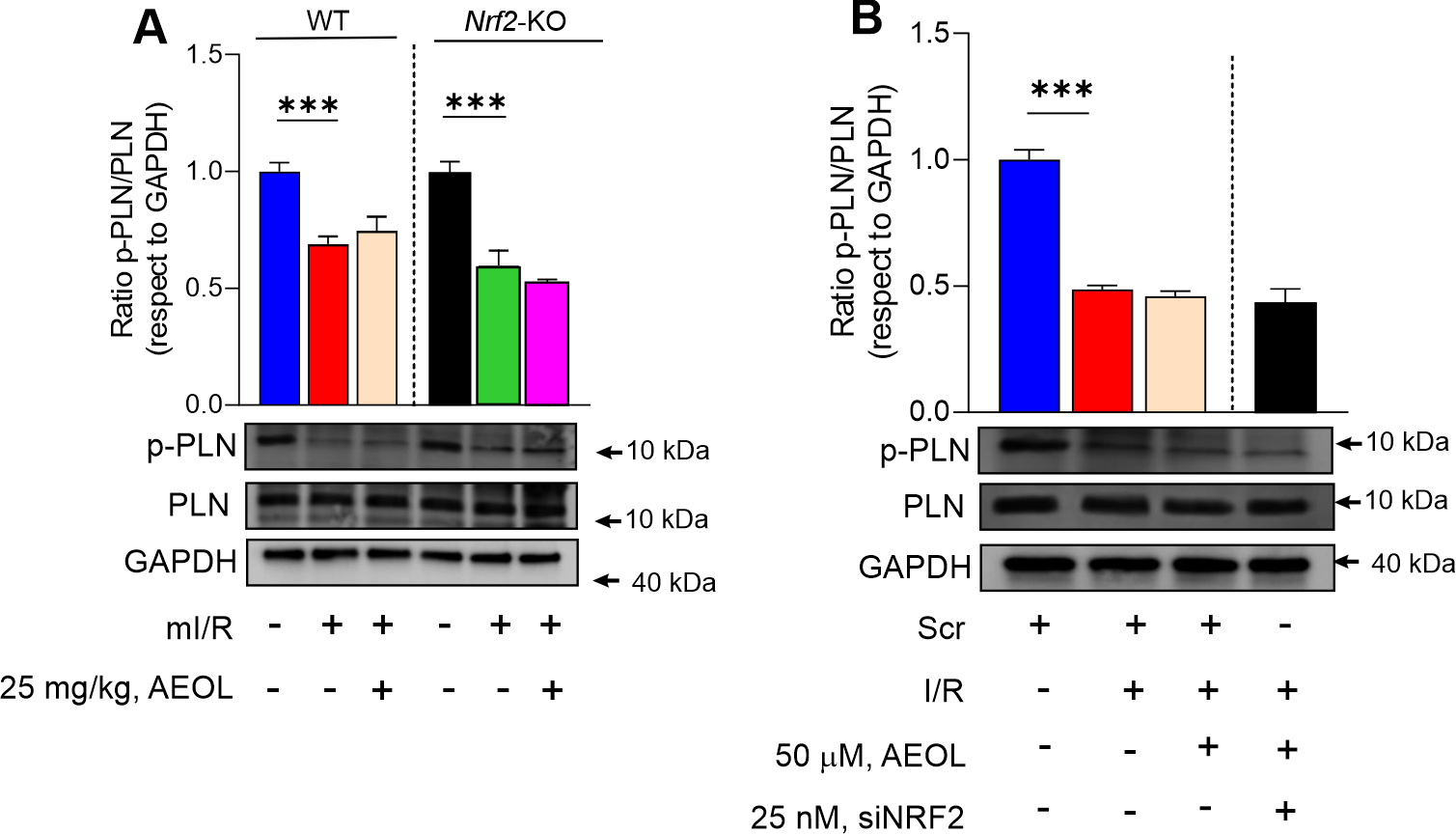
**7.-

**Figure S7.** *AEOL treatment does not affect PLN phosphorylation.* (A) Phospho-PLN/total PLN ratio in the infarct border zone of wild type and *Nrf2*-KO mice 24 h after mI/R. Mice were treated with AEOL or vehicle (0.9% NaCl) 15 min after reperfusion onset. (B) PLN/total PLN ratio in hiPSCMs 24 h after simulated mI/R. Cells were treated with AEOL at the onset of reoxygenation. Data were obtained from a sample size of n=7 mice per group for *in vivo* assays and n=5 independent assays per group for *in vitro* procedures and are presented as mean ± SEM. ***p < 0.001, determined by one-way ANOVA followed by post hoc Bonferroni correction. Abbreviations are defined in the abbreviations list.

8.-

Here, full-length, uncropped representative Western blot images are presented, illustrating the experimental findings. For in vivo experiments, data were obtained from n=7 mice per experimental group, while in vitro experiments were based on samples from n=5 independent assays per group. Each sample was analyzed in duplicate to ensure data consistency and reliability. The selected images represent the overall Western blots performed and analyzed throughout the study. It is important to note that, in some instances, the full images provided include one of the assay replicates, which contributed to the total dataset analyzed. The datasets used and/or analyzed for the western blot analyses during the current study are available from the corresponding author upon reasonable request.

1. **
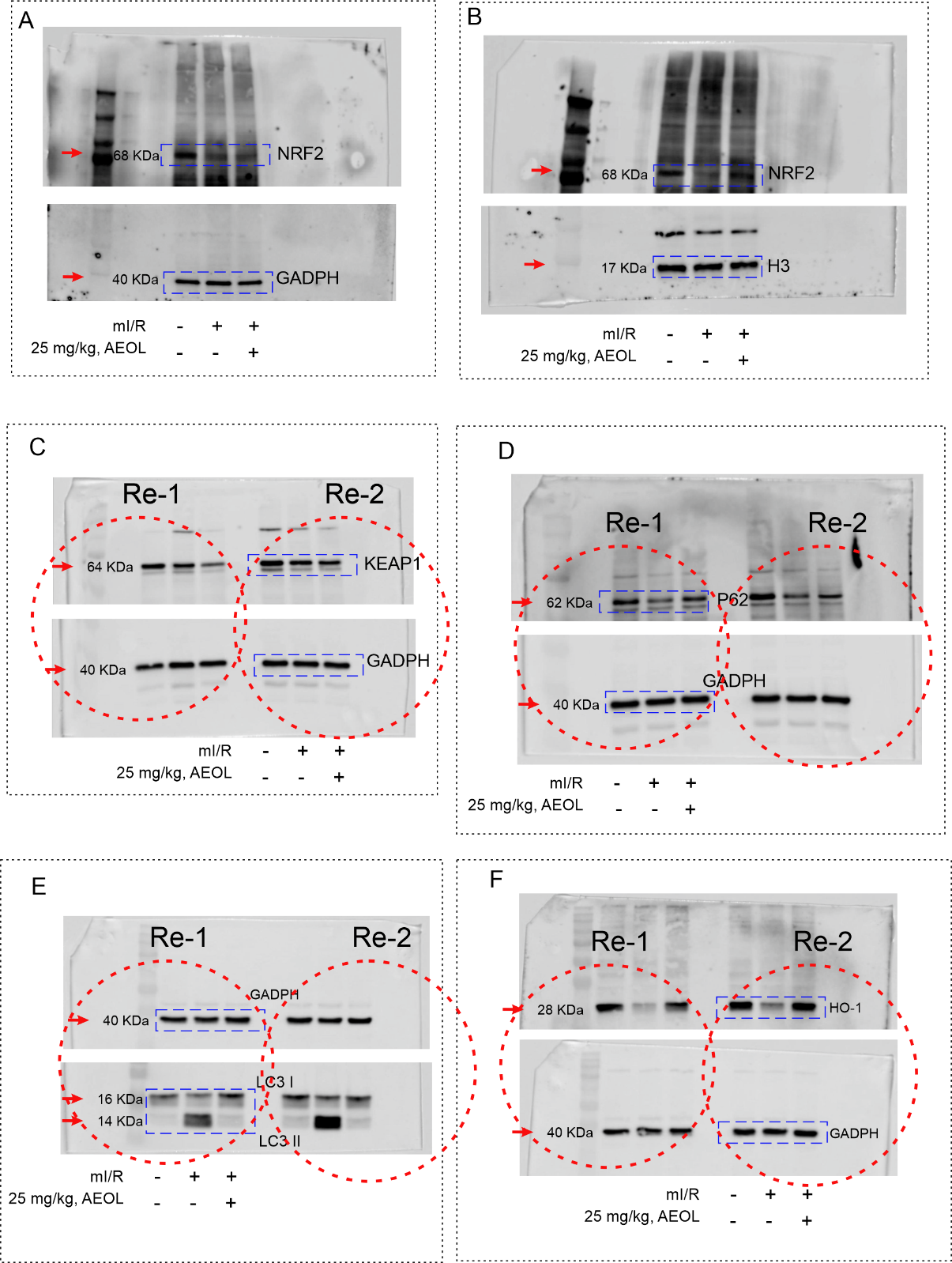
**Representative western blot images selected for figure 4.

**Figure S8.**

Representative full Western blot images for Figure 4. The blots were cropped from different parts of the same gel, with some samples duplicated within the same blot. Cropped areas are marked with a discontinuous blue line. The red arrow highlights the position of the band of interest. Additionally, the experimental procedure for each lane is indicated. The red dashed circles indicate two of the replicates from the total performed in the study. Abbreviations: Re: replicate. Other abbreviations are defined in the main manuscript

1. Representative western blot images selected for figure 6.

**
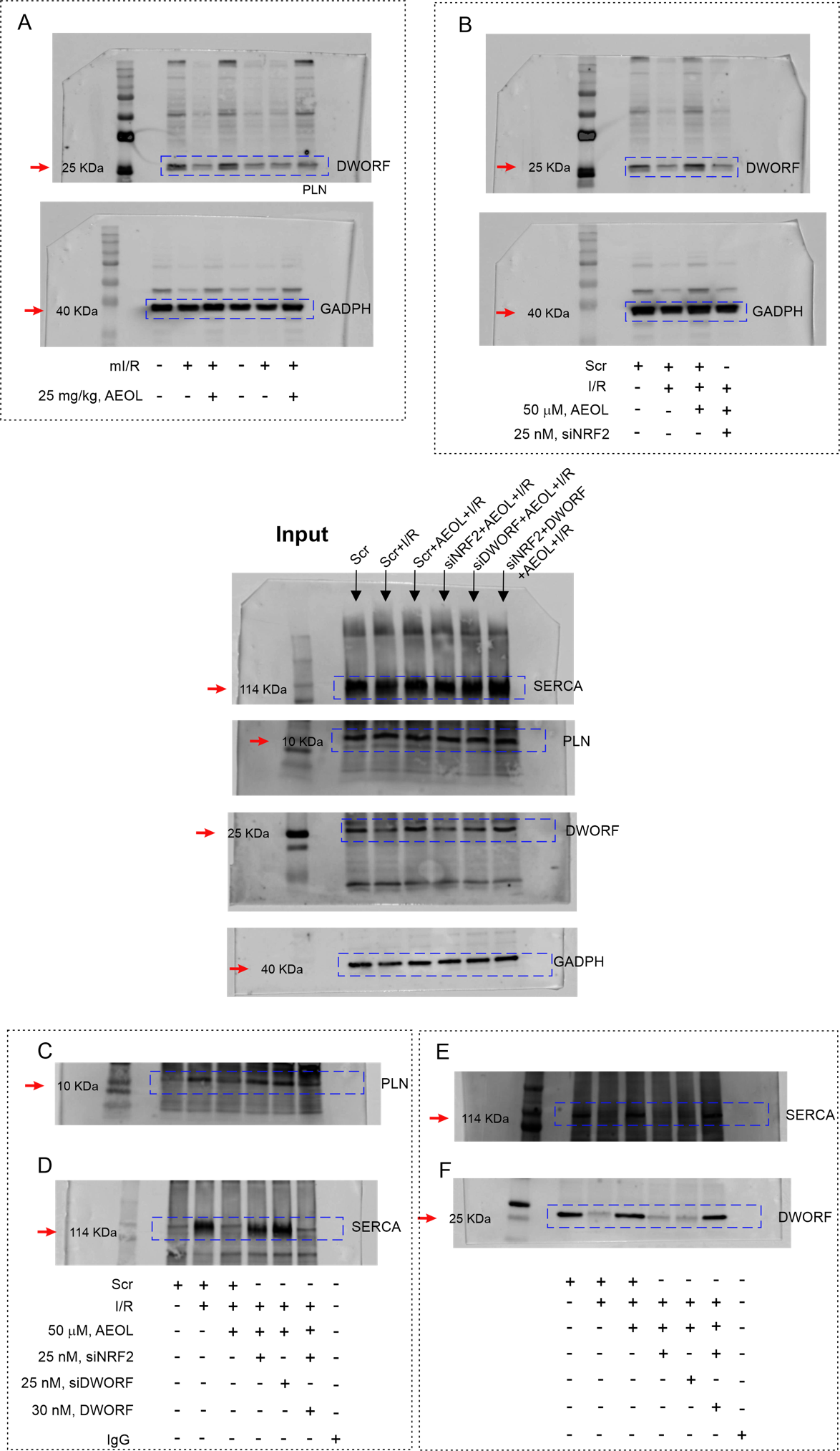
**

**Figure S9.**

Representative full Western blot images for Figure 6. The blots were cropped from different regions of the same gel, with cropped areas indicated by a discontinuous blue line. The red arrows highlight the position of the bands of interest. Additionally, the experimental procedure for each lane is indicated.

1. Representative western blot images selected for figure 6.


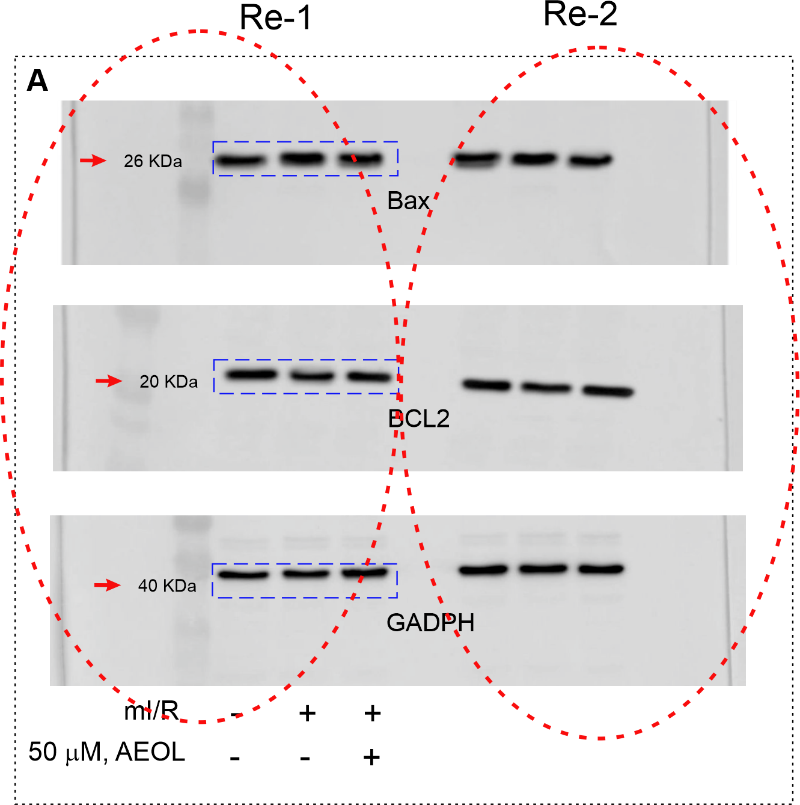


**Figure S10.**

Representative full Western blot images for Figure S5. The blots were cropped from different regions of the same gel, with cropped areas indicated by a discontinuous blue line. One of the analyzed samples is shown as a duplicate within the same blot. The red arrows highlight the position of the bands of interest. Additionally, the experimental procedure for each lane is indicated. The red dashed circles indicate two of the replicates from the total performed in the study. Abbreviations: Re, replicate. Other abbreviations are defined in the main manuscript.

1. **
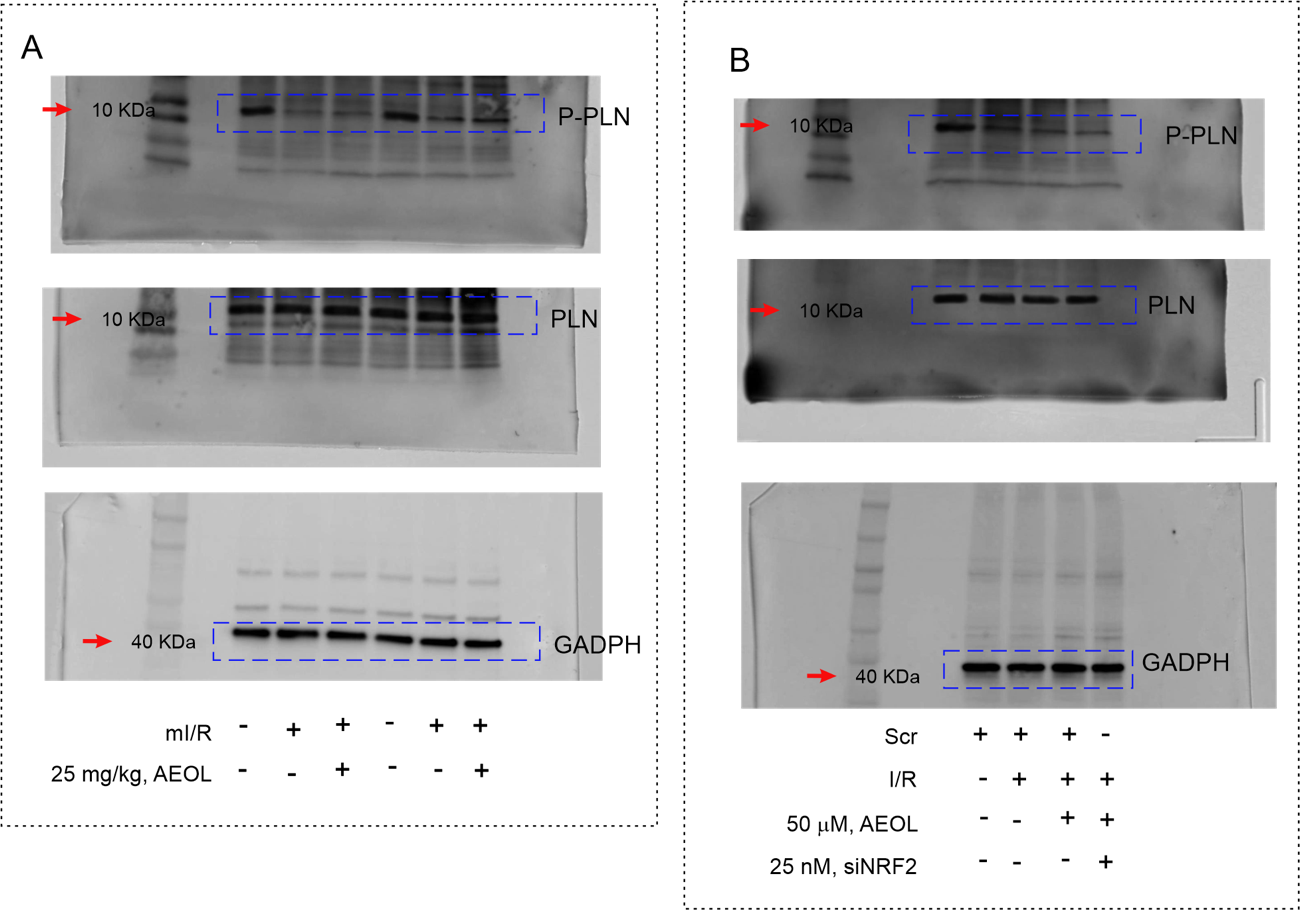
**Representative western blot images selected for figure 6.

**Figure S11.**

Representative full Western blot images for Figure S7. The blots were cropped from different regions of the same gel, with cropped areas indicated by a discontinuous blue line. The red arrows highlight the position of the bands of interest. Additionally, the experimental procedure for each lane is indicated.

**References**

1. Asensio-Lopez MC, Lax A, Fernandez del Palacio MJ, et al. Yin-Yang 1 transcription factor modulates ST2 expression during adverse cardiac remodeling post-myocardial infarction. J Mol Cell Cardiol. 2019; 130: 216–33.

2. Smith PK, Krohn RI, Hermanson GT, et al. Measurement of protein using bicinchoninic acid. Anal Biochem. 1985; 150: 76–85.
